# Supplementary material for: Absolute energy level positions in tin- and lead-based halide perovskites
Source: Nat Commun. 2019 Jun 12;10:2560. doi: 10.1038/s41467-019-10468-7 (PMC6561953; doi:10.1038/s41467-019-10468-7)
Supplement: Supplementary file 1 — Supplementary Information [file 41467_2019_10468_MOESM1_ESM.pdf]

Supplementary Information

Absolute energy level positions in tin and lead based halide perovskites

S.Tao *et al.*

Content

1. Supplementary Tables and Figures *Page 2*
   - *Comparison of changes in energy levels*
   - *Semi-core states*
   - *Calculated Goldschmidt's tolerance factors*
   - *Tight-binding parameters*
2. Supplementary Methods *Page 4*
   - *Details of DFT calculations*
   - *Broadening and fitting procedure for the DFT calculated DOS*
   - *Procedures used on the experimentally measured DOS*
   - *Calculation of ionization energy and electron affinity*
   - *Description of additional measurement techniques*
   - *Sample preparation protocols*
3. Supplementary Notes and Figures *Page14*
   - *Comparisons between measured and DFT calculated densities of states*
   - *Supplementary Material Datasets (XRD, SEM, XPS, UV-vis)*
4. Supplementary Discussion *Page 38*
   - *Experimental sample-to-sample variation*
   - *Changes in ionization energy with different compositions*
5. Supplementary References *Page* 41

1. Supplementary Tables and Figures

Comparison of changes in energy levels

In the main manuscript the trends in energy level positions are shown sorted by the size of the optical gap; here, in Supplementary Figure 1, this graph is re-plotted and in addition similar sketches are presented in which the materials are sorted by their IE or EA values, respectively.

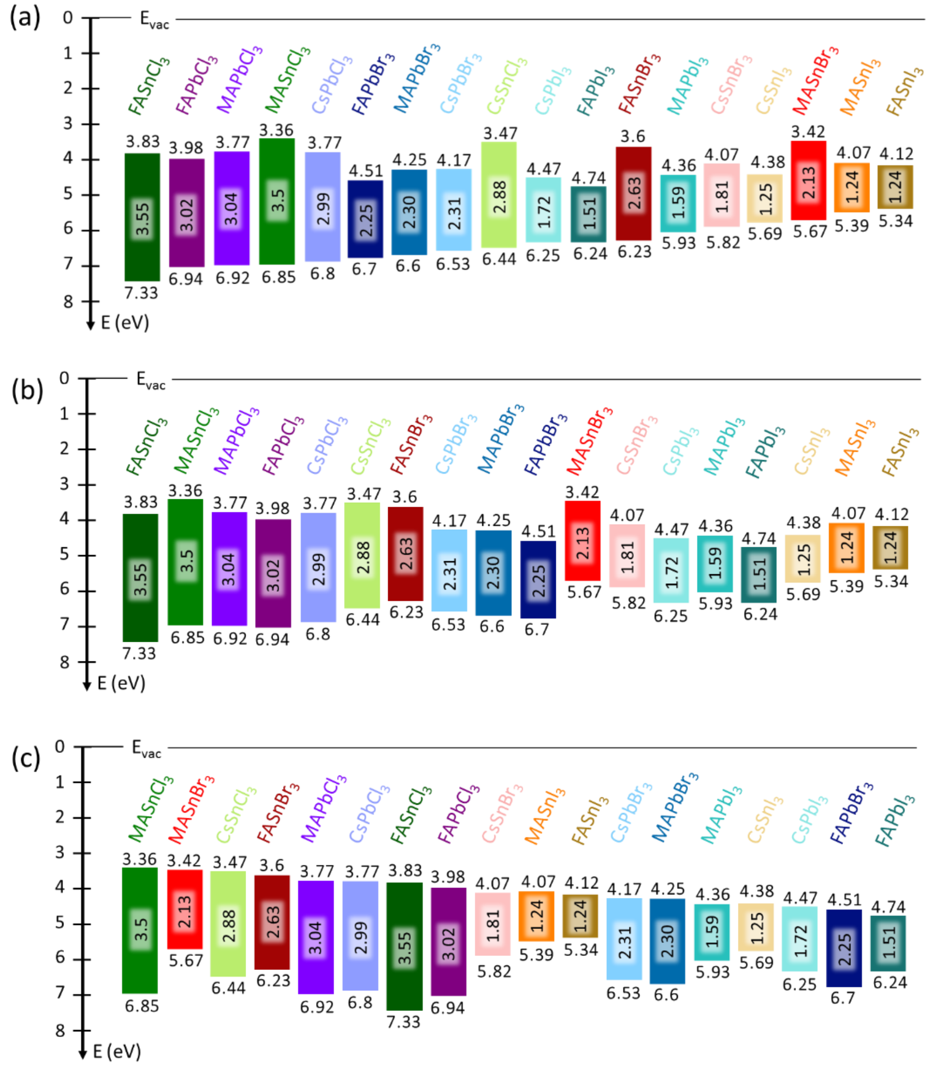

Supplementary Figure 1 | Graphical representation of IE, EA, and band gap data of the perovskites, ordered with respect to (a) decreasing band gap, (b) decreasing IE, and (c) increasing EA value.

Semi-core states

As mentioned in the main article and shown there in Figure 1, in the experimental studies we find that features originating from the A cation have approximately the same energetic position with respect to the vacuum level, irrespective of the perovskite composition. This is used, for instance, to identify well-prepared films (in addition to the stoichiometric information obtained by XPS). This result is also found in our DFT analysis of $CsMX_3$, where the Cs 5*s* and 5*p* semi-core states for different halides X lie within ± 0.1 eV as shown in the Supplementary Table 1 below.

Only for $CsSnCl_3$, a somewhat larger variation of ~0.3 eV is found; this material shows more deviation in the experimental study as well.

Supplementary Table 1 | Energies (in eV) of Cs 5s and 5p semi-core states

Pb

	**I**	**Br**	**Cl**
Cs *s*	-26.57	-26.59	-26.46
Cs *p*	-13.34	-13.38	-13.27

Sn

	**I**	**Br**	**Cl**
Cs *s*	-26.54	-26.47	-26.78
Cs *p*	-13.32	-13.27	-13.63

Values are obtained from DFT calculations on Cs containing perovskites with cubic structure. The values are shifted such that the VBM position matches the position of the experimental IE.

Calculated Goldschmidt's tolerance factors

Goldschmidt's tolerance factor is used as an indicator for the stability of perovskite crystal structures (AMX_3), and their tendency to distort; it is defined as: $TF = \frac{r_A + r_X}{\sqrt{2}(r_M + r_X)}$, with $r_\square$ the radius of the ions. Supplementary Table 2 lists the extracted tolerance factors of the perovskite under investigation here. For the ionic radii we use Shannon's crystal radii. The radii of 1.81, 2.70, 2.79 Å are used for Cs^+, MA^+, and FA^+, respectively; 1.20 and 1.33 Å are used for Sn^{2+} and Pb^{2+}, respectively; 1.67, 1.82 and 2.06 Å are used for Cl^-, Br^-, and I^-, respectively. [values taken from Ref. 1]

Supplementary Table 2 | Calculated tolerance factors for the different perovskites

Pb

	**I**	**Br**	**Cl**	
TF	0.81	0.82	0.82	**Cs**
TF	0.99	1.02	1.03	**MA**
TF	1.01	1.04	1.05	**FA**

Sn

	**I**	**Br**	**Cl**	
TF	0.84	0.85	0.86	**Cs**
TF	1.03	1.06	1.08	**MA**
TF	1.05	1.08	1.10	**FA**

Tight-binding parameters

Supplementary Table 3 | Tight-binding parameters (in eV relative to the vacuum level)

	$CsPbI_3$	$CsPbBr_3$	$CsPbCl_3$	$CsSnI_3$	$CsSnBr_3$	$CsSnCl_3$
E_1^{exp}	-4.53	-4.22	-3.81	-4.44	-4.07	-3.56
E_2^{exp}	-6.25	-6.53	-6.80	-5.69	-5.85	-6.44
E_3	-14.51	-14.88	-15.11	-14.04	-14.39	-15.24
E_4	-18.71	-21.47	-21.94	-18.87	-21.56	-22.52
$E_{X,s}$	-18.44	-20.99	-21.19	-18.66	-21.39	-22.31
$E_{X,p}$	-8.15	-9.05	-9.79	-8.28	-9.11	-10.35
$E_{M,s}$	-12.61	-12.36	-12.12	-11.45	-11.13	-11.33
$E_{M,p}$	-4.80	-4.70	-4.56	-4.65	-4.24	-3.77
$V_{M,p\text{-}X,s}$	1.01	1.47	1.86	0.87	0.88	0.99
$V_{M,s\text{-}X,p}$	1.00	1.11	1.15	1.11	1.21	1.26

$E_{X,s}$, $E_{X,p}$, $E_{M,s}$, $E_{M,p}$, are the on-site *s* and *p* atomic energy levels of X anions and M cations, respectively; $V_{M,p\text{-}X,s}$, $V_{M,s\text{-}X,p}$ are the hybridization strengths (hopping parameters) between the indicated orbitals. E_1^{exp} and E_2^{exp} are specific energies of the conduction and valence band onsets (CBM and VBM), as extracted from experimental electron affinity and ionization energy, respectively; E_3 and E_4 are energy levels within the valence band, extracted from the DFT calculations (see main text). $E_{M,p}$ includes the downward shift of -2/3 Δsoc due to the spin-orbit coupling, with 2/3 Δsoc = 1.1 eV for Pb and 2/3 Δsoc = 0.4 eV for Sn.

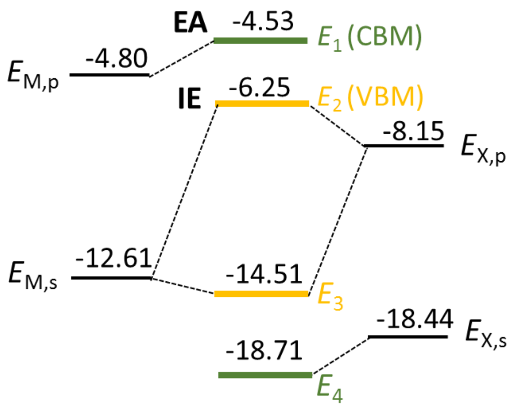

Supplementary Figure 2 | Scheme of the energy levels involved in the tight-binding analysis, using cubic $CsPbI_3$ as a model system. $E_{X,s}$, $E_{X,p}$ and E_1, E_2, E_3, and E_4 are derived from DFT band structure calculations. $E_{M,p}$ and $E_{M,s}$ (listed in Table 3 above) are derived from the tight-binding model. Note, E_1 and E_2 are set to the experimental values of the electron affinity and the ionization energy; the DFT derived values of $E_{X,s}$, $E_{X,p}$, E_3, and E_4 are shifted accordingly.

2. Supplementary Methods

Details of DFT calculations

To compare the experimental DOSs to the DFT calculated ones, it is important to take into account the structural disorder of metal-halide perovskites at finite temperature in the structural models in DFT calculations. The lattice of the metal halide perovskites is proven to be highly dynamic due to its soft nature. Dynamic disorder of inorganic frameworks and of organic or inorganic cations at finite temperature was observed in previous computational[2,3] and experimental studies[4,5]. In order to model this disorder in a simple way, we adopt a tetragonal cell, consisting of 8 AMX_3 units, for organic-cation-containing perovskites (Supplementary Figure 3 (a)) and an orthorhombic cell, consisting of 4 AMX_3 units, for Cs-containing perovskites (Supplementary Figure 3 (b)). These cells are (i) sufficiently large to model some of the disorder seen in both A cation (MA, FA, and Cs) and the MX_3 inorganic framework, and (ii) they are not too large, so that we can afford DFT calculations using a hybrid functional and take spin-orbit-coupling into account. This way, as shown from our comparisons of DFT and experimental DOSs of all 18 perovskites (Figure 2 in main text and Supplementary Figure 6 here), excellent agreement in the occupied DOSs is found. We note that smaller unit cells are insufficient to describe the DOS realistically due to an unrealistic distribution (in case of Cs) and uniform orientation (in cases of MA and FA) of A cations.

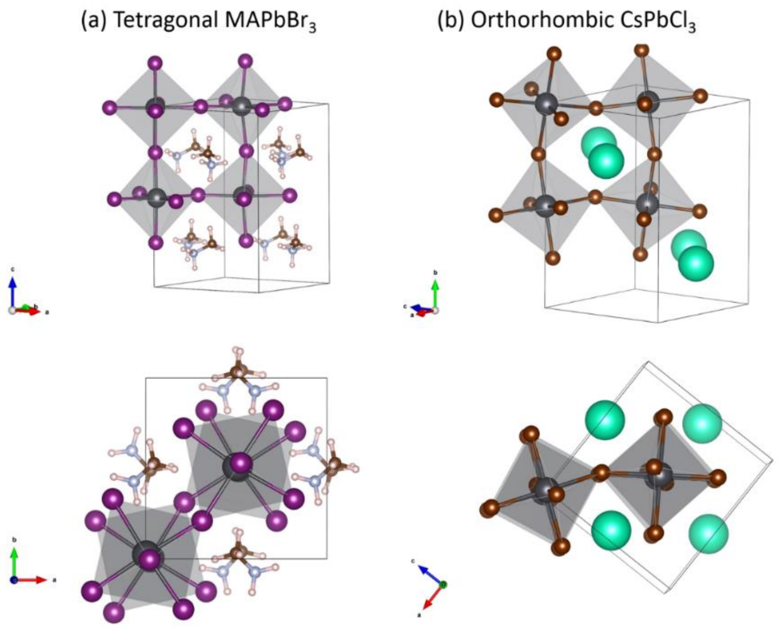

Supplementary Figure 3 | Structural models used for DOS calculations: tetragonal cell for $MAPbBr_3$ and orthorhombic cell for $CsPbCl_3$.

To further motivate the choice of crystal structures employed here, Supplementary Figure 4 shows a comparison between the calculated DOS in a primitive cubic cell vs. the tetragonal cell of Supplementary Figure 3(a) for $MAPbBr_3$, as well as a primitive cubic cell vs. the orthorhombic cell of Supplementary Figure 3(b) for $CsPbCl_3$. XRD measurements show that both of these systems form a cubic crystal structure at room temperature (see Supplementary Notes 1 to 18 for XRD measurements). However, if a primitive cubic unit cell is used to calculate the DOS, significant deviations from experiments are found. In particular, features at the band edges are missing. Another important discrepancy found in cubic $MAPbBr_3$ is that the second peak deeper in the VB (around 3 to 4 eV, marked by grey arrow) shifts to the left compared to the one found in a tetragonal cell. Again, this shift leads to a significant inconsistency with the experimental DOS. We attribute this to the uniform orientation of MA ions (ferroelectric alignment), enforced when using a primitive cell, which is unlikely to be present at room temperature due to the rotational motions of the MA ions. Highly ordered MA cations create an electrostatic field that induces a shift of the Br and Pb atomic levels, which modifies the band structure. It can, therefore, be concluded that, even though these systems do not show lattice distortion or structural disorder in the XRD measurements, there is nonetheless a dynamic disorder in the MX_3 lattice, which renders these systems only cubic on average. In our calculations, we can only represent this dynamic disorder by a static variation in the orientation of the MA ions. Our tetragonal cell containing 8 MA/FA molecules orthorhombic cells with 4 Cs are the smallest one that approximately capture the spread in these orientations.

Based on the above analysis, we have adopted tetragonal structures for all MA and FA perovskites and orthorhombic structures for Cs perovskites. Structural models for $MAPbBr_3$ and $CsPbCl_3$ are found in Supplementary Figure 3. We use the experimental XRD data of $MAPbI_3$ and $CsSnI_3$ as starting point, and optimize all (tetragonal and orthorhombic) structures of the 18 perovskites using the same DFT settings. It should be noted that slight overestimation or underestimation of lattice parameters in the DFT calculations will add a small uncertainty to the calculated energy levels near band edges and slightly alter the band gaps (due to a change of hybridization strength between M cations and I anions, see the main text). However, varying the size of cells in the DFT calculations by 1-2% only leads to small changes in band gaps in the range of 30 - 60 meV [6]. Another source of uncertainty is introduced by temperature effects, which are not taken into account in these DFT calculations. Experiments show that when the temperature is varied by 150 K, changes in band gap in the range of 30-60 meV are found [7]. Overall, the variations introduced by lattice size and temperature are therefore smaller than the experimental uncertainties and sample-to-sample variations (main text Table 1). Mostly importantly, our fitting procedure only makes use of the shape and relative

positions of main peaks (Figure 2 in main manuscript). These remain almost unchanged. So we can disregard the differences introduced by the small changes in lattice parameters.

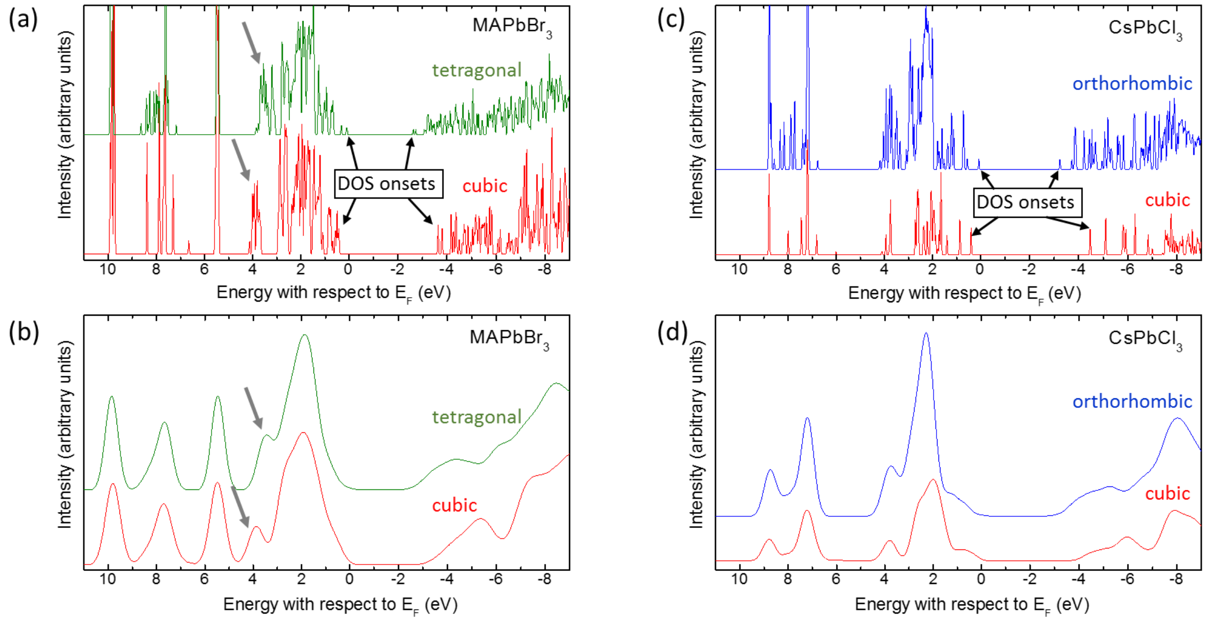

Supplementary Figure 4 | Comparison between the DFT-calculated DOSs using different structures. (a) shows the calculated DOS and (b) shows the broadened DOS of $MAPbBr_3$, using a primitive cubic unit cell (red curve), and a tetragonal supercell (green curve). The structural motive of the latter is shown in Supplementary Figure 3. In (c) and (d) the same comparison is made for the DOSs of $CsPbCl_3$, using a primitive cubic vs. an orthorhombic supercell. The procedure for peak broadening is described below. Here, for a direct comparison to experiments, the signs of the energy levels are adjusted to the experimental ones, i.e., positive values for VB energies and negative values for CB energies.

Broadening and fitting procedure for the DFT calculated DOS

A) Treatment of unbroadened DFT spectra

The calculated DOSs, shown in Figure 2 of the main article and in Supplementary Figure 6, are plotted with respect to Fermi level and for a direct comparison with experiments, the sign of the energy levels are adjusted to the experimental ones, i.e. the positive values for VB and negative values for CB bands are used. For halide perovskites, the standard DFT functional PBE gives a DOS that needs to be stretched to match photoemission data. To partly correct for this, it is common to apply a hybrid functional to calculate the DOS [8], as the Hartree-Fock contribution to such a functional stretches the spectrum. We use the PBE0 hybrid functional, which we find has a similar performance as the HSE functional used in Ref. [8], i.e., the band widths in halide perovskites are a few percent too small. Comparing theory and experiment, we are able to determine the necessary stretching to achieve a best fit. The stretching factors for conduction and valence band regions are always kept the same and are at most 8% (listed in Supplementary Table 4), which is similar to previous reports on $MAPbI_3$ and $CsPbBr_3$ [8].

Supplementary Table 4: Stretching factors used on calculated DFT spectra to achieve agreement with experiment

material	*$CsPbI_3$*	*$MAPbI_3$*	*$FAPbI_3$*	*$CsPbBr_3$*	*$MAPbBr_3$*	*$FAPbBr_3$*	*$CsPbCl_3$*	*$MAPbCl_3$*	*$FAPbCl_3$*
stretching	1.06	1.08	1.08	1	1.02	1.05	1.06	1.04	1.06
material	*$CsSnI_3$*	*$MASnI_3$*	*$FASnI_3$*	*$CsSnBr_3$*	*$MASnBr_3$*	*$FASnBr_3$*	*$CsSnCl_3$*	*$MASnCl_3$*	*$FASnCl_3$*
stretching	1	1	1.04	1	1.03	1.02	1	1	1

Neither pure DFT functionals such as PBE, nor hybrid functionals such as PBE0 or HSE give very good band gaps. PBE0 actually gives band gaps that are too large, whereas PBE and HSE give band gaps that are too small, see Ref. [8] . Since we are mainly interested in a realistic representation of the DOS, we used the PBE0 hybrid functional and correct the DFT gap such that it coincides with the experimental optical gap. This was achieved by shifting the CB DOS while keeping the VB constant. As discussed in the main article, this treatment can induce an error equal to the exciton binding energy. However, as this value is typically small, this will not significantly affect the extracted energy level values.

B) Broadening of DFT spectra

Next, we broaden the DFT spectra according to the experimental resolution. This is done separately for the VB and CB region, since the resolutions of UPS (VB) and IPES (CB) differ.

A Gaussian function

$$g(x) = \frac{1}{\sqrt{2\pi\sigma^2}} e^{-(x-\mu)^2/2\sigma^2} \quad (1)$$

(μ is the mean value, and σ is the variance) is used on each data point of the spectrum to broaden the features. For the VB region, a value of σ = 0.26 is used and for the CB region σ = 0.34. Supplementary Figure 5a shows examples of Gaussian normal distributions for μ = 0 and various values of σ. Supplementary Figure 5 b shows as examples the calculated VB DOS of $FAPbI_3$ (black curve) and the same DOS convoluted with a Gaussian function for different values of σ.

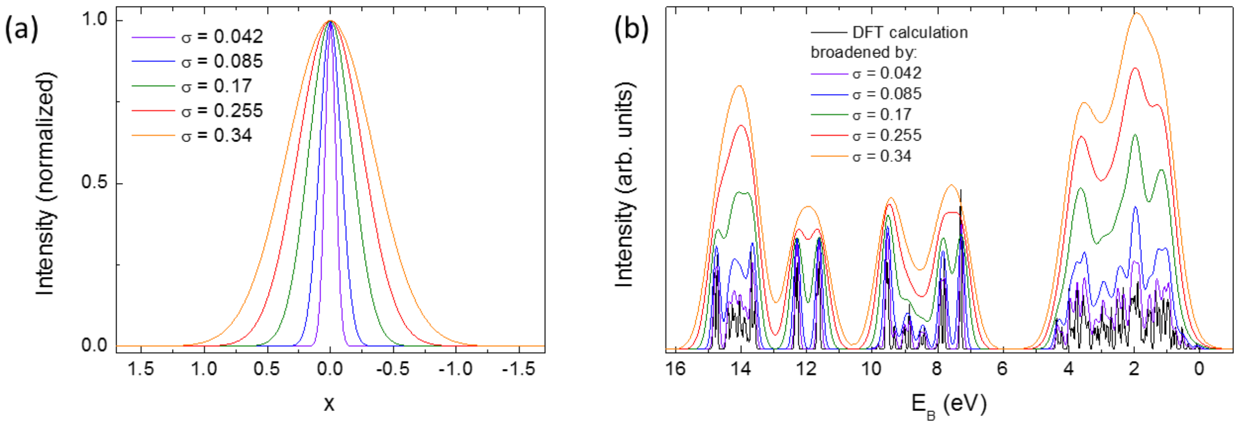

Supplementary Figure 5 | Examples visualizing the Gaussian broadening. a) Gaussian peaks with various broadening. b) example of the theoretical VB DOS of $FAPbI_3$, as calculated (black) and with various broadenings as indicated in the legend.

C.) Fitting of the DFT calculated DOS

In order to reliably correlate the calculated to the experimental DOS, as shown for $FAPbI_3$ and $FASnI_3$ in Figure 2 of the main article and for all 18 perovskites in Supplementary Figure 6 a) - r) below, the calculated DOSs are fitted with a set of Gaussian peaks; these are in principle random, but are chosen in such a way that they describe the different perovskites in a consistent way. While the valence band is fitted over a wide energy range (to approximately 10 eV below the VBM), only the first feature above the CBM is fitted for the CB. This was done since the agreement in the unoccupied DOS between theory and measurement is not sufficient to draw any conclusion from states higher up in the CB.

Procedures used on the experimentally measured DOS

A) Fitting of the experimentally measured DOS

Next, a similar set of Gaussian peaks is used to fit the experimental DOS in order to be able to align the experimental with the theoretical spectrum as already briefly elaborated in the main text. Fixed distances between the Gaussian

peaks, as extracted from the theoretical fit, are employed, while the Full Width Half Max (FWHM) is used as a fitting parameter (this value was then kept constant for different samples of the same material). In the occupied DOS (UPS measurement) good agreement is found between theory and experiment, as shown throughout Supplementary Figure 6. Here, for each of the 18 perovskites, three separate and representative measurements are used. In analogy to Figure 2 from the main article, the different colored bars act as guide for the eye to show how different features align and match. Of course intensities of the individual features do not necessarily agree, since they depend on the cross section of the incident light with the material, and therefore on the photon energy.
In the energy region deeper than 6 eV, we find that the theoretical and experimental data deviate. In this energy region we find, for instance, states of the A cation that do not hybridize with the MX_3 frame, such as the Cs-related feature in Figure 3 in the main text. These states are then localized on the A cation. At deeper energies one also finds semicore states of the B cations and X anions, which hybridize little with their environment, and are more localized than the valence states. Standard functionals, either pure density functionals such as PBE, or hybrid functionals such as PBE0, do not give the energy levels of localized states very well, because of the electron self-interaction error associated with these functionals [9]. These localized states are however not relevant for the DOS closer to the VBM. It is important to note that in some cases features at the VB are either broadened compared to theory or there is actually the need to insert an additional peak. This is most notable for $CsPbI_3$, $MASnI_3$, $CsSnBr_3$, $MAPbBr_3$, and $FAPbBr_3$. Such differences can, for example, be introduced by deviations in surface composition, i.e. a specific surface termination. Examples of changes in the shape of the DOS with variations in bulk composition are discussed below in in the Section 4: Supplementary Discussion. In addition, defect states can also induce states at the band edges; one should not forget that UPS is a very surface sensitive technique (probing depth around 1-2 nm) and a surface is always a defect. Such states have been reported to reduce the surface electronic band gap even for cleaved single crystals [10]. This surface effect is less pronounced when higher energy photons are used for excitation since this increases the probing depth [11]. By fitting our measurement to the DFT data, we are however not affected by these states and can distinguish them from the VB and CB DOS. As a further remark to the occupied DOS, the FA cation has a feature that is present only a few eV below the VB onset; its position is indicated as feature D in Figure 1 (main article) and highlighted as a shaded grey peak in the plots in Supplementary Figure 6. Comparing theory and experiment here, it is obvious that DFT misplaces this feature slightly, which is again due to the more localized nature of this state, which is, of course, localized on the FA cation. In experiment, its position can be placed with high accuracy by comparing the density of states of FA containing species with their MA/Cs counterparts which do not have a contribution by the A-site cation in this region.
Regarding the unoccupied DOS, a similar Gauss fitting procedure is tried for the CB region. As discussed in the main article this turns out to be challenging. We would only like to note here that interestingly, Sn based perovskites always show a much lower intensity at the CBM compared to the Pb analogs. Often the onset is barely visible and hard to distinguish from the background making it absolutely necessary to have the theoretical data to find the correct onset.

Calculation of ionization energy and electron affinity

The VB and CB onsets (i.e. E_2^{exp} and E_1^{exp}) are determined by the position of the first feature in the unbroadened DFT-DOS. Since the spectra have been aligned, as discussed in the previous section, the value can be read out from the dashed line in Supplementary Figure 6. In practice, the value is extracted by determining the distance between the main peak (for example green shaded area for occupied DOS) and the unbroadened VB onset in the theoretical data set. This offset is then added to the peak values found in the measurement. To calculate the IE and EA, the work function has to be included:

$$IE = Wf + E_2^{\text{exp}}, \tag{2}$$

$$EA = Wf - E_1^{\text{exp}} \tag{3}$$

The values of Wf, IE, and EA for of all samples used for the evaluation (3 samples per material) are listed in Table 6.

Description of additional measurement techniques

Various additional measurement techniques were used to characterize the perovskite films, especially during the initial optimization steps of material preparation. The resulting measurements are shown and shortly discussed in the Supplementary Notes 1 to 18 in Section 3: Supplementary Notes and Figures.

Absorption measurements (UV-vis): Absorption measurements were performed in air using a Cary 50 UV-Vis spectrometer from Varian in ranges from 250 - 1100 nm with a scan rate of 60 nm/min. The spectrum of a reference sample, corresponding to the substrate (glass/ PEDOT:PSS), was subtracted from each measurement.

X-ray photoelectron spectroscopy (XPS): XPS measurements were performed in the same setup as UPS, using also the hemispherical electron energy analyzer (Phoibos 100, Specs). For excitation, a non-monochromatic x-ray source with Mg anode was used, having a photon energy of hν = 1253.6 eV. The survey scans are taken with a pass energy of 70 eV and the detailed peaks with a pass energy of 10 eV; here, the energy resolution is approximately 800 meV. Peak fits of the XPS peaks were done using the program *XPS Peak Fit 4.1* and film stoichiometry was determined by evaluating the individual peak areas and correcting those with the relative sensitivity factors (RSF). These RSF factors were calibrated for this system relative to carbon; the values used are RSF(N *1s*) = 1.8, RSF(Cs $3d_{5/2}$) = 40.3, RSF(I $3d_{5/2}$) = 32.8, RSF(Br $3d_{5/2}$) = 2.13, RSF(Cl $2p_{3/2}$) = 1.8, RSF(Pb $4f_{7/2}$) = 16.5, and RSF(Sn $3d_{5/2}$) = 24.

X-ray diffraction (XRD): XRD spectra were recorded using a Panalytical Empyrean system with an excitation via a Cu K_{α} anode (λ = 1.54056 Å); the angle 2Θ was scanned between 10 and 40° with a step width of 0.0131°. For most of the measurements Glass/PEDOT:PSS was used as substrate, while in some cases Glass/ITO/PEDOT:PSS was used. The latter ones produce additional reflexes from the ITO layer, which are indicated in the measurements in the Supplementary Notes 1 to 18. The measurements were performed in air. Here, to prevent degradation, the sensitive perovskite layers (e.g. tin-based ones or $CsPbI_3$/$FAPbI_3$) were coated with PMMA (30 mg/ml in Toluene, spin coating at 3000 rpm for 30 s) to form a protective barrier layer on top.

Scanning electron microscopy (SEM): SEM measurements were performed in a Zeiss Neon-40 system, images shown here are taken with an In-lens detector. Images of different magnifications are shown, since samples showed structures on rather different length scales.

Sample preparation protocols

All perovskite films throughout this work are prepared under N_2 atmosphere (for solution processing) or in ultra-high vacuum (for thermal evaporation). Samples were given different names (i.e. $MAPbI_3$ S1, $CsPbBr_3$ S2, …) depending on the preparation protocol used, as listed in the Supplementary Table 6 below. It should, however, be noted that samples called e.g. S1 throughout the article are not necessarily identical samples, it only means they were similarly prepared (usually separate samples for PES, XRD, and UV-vis were fabricated). Exceptions are the UPS/IPES/XPS measurements that are always done together on the same sample.

For UP, IPES, XPS, and SEM investigations, PEDOT:PSS covered ITO substrates were used while mostly glass substrates covered with PEDOT:PSS were employed for UV-Vis and XRD measurements. The filtered PEDOT:PSS dispersion (Clevios P VP Al 4083 from Heraeus) was deposited on top by a two-step spin coating procedure, spinning first for 25 s at 4000 rpm and then 5 s at 4000 rpm to yield about 40 nm thick layers. The PEDOT:PSS films were annealed at 150 °C for 10 min. After that, the substrates were transferred into the nitrogen atmosphere.

The vendors for the solvents and materials used for perovskite preparation are listed in Supplementary Table 5 while the processing procedure are listed together with extracted values of work function (Wf), ionization energy (IE), electron affinity (EA), and XPS determined film composition in Supplementary Table 6.

Supplementary Table 5 | List of used solvents and precursors and their suppliers.

Formula	Chemical	supplier	purity
DMF	N N-Dimethylformamide, HPLC grade	Sigma-Aldrich	≥ 99.9%
DMSO	Dimethyl Sulfoxide, analytical reagent grade	Fisher Scientific	≥ 99.9%
CB	Chlorobenzene for HPLC	Sigma-Aldrich	99.9%
Tol	Toluene for HPLC	Sigma-Aldrich	≥ 99.9%
ODCB	1,2-Dichlorobenzene	Sigma-Aldrich	99%
IPA	2-Propanol CHROMASOLV LC-MS	Honeywell	99.9%
CsI	Cesium iodide	Sigma-Aldrich	99.999%
CsBr	Cesium Bromide	TCI	>99.0%
CsCl	Cesium Chloride	TCI	>99.0%
PbI_2	Lead(II) iodide trace metals basis	Sigma-Aldrich	99.999%
$PbBr_2$	Lead(II) bromide, trace metals basis	Sigma-Aldrich	99.999%
$PbCl_2$	Lead(II) chloride, anhydrous	Sigma-Aldrich	99.999%
SnI_2	Tin(II) iodide, trace metals basis	Sigma-Aldrich	99.99%
$SnBr_2$	Tin(II) bromide	Aldrich	
$SnCl_2$	Tin(II) chloride, trace metals basis	Sigma-Aldrich	≥ 99.99%
NH_4Cl	Ammonium Chloride, Suprapur	EMD Millipore	99.995%
MA I/Br/Cl<br>FA I/Br/Cl	Methylammonium and formamidinium salts were prepared in house using a standard synthesis procedure		

Supplementary Table 6 | Sample preparation protocols and extracted values of work function (Wf), ionization energy (IE), electron affinity (EA), and XPS determined film composition.

Sample	Preparation (precursor, concentration, solvent, processing, annealing)					Wf [eV]	IE [eV]	EA [eV]	XPS stoichiometry
$CsSnI_3$ S1	CsI and SnI_2	0.5 M	DMF	Spin coating: 30s at 5000 rpm	10 min at 100 °C	5.02	5.70	4.44	Cs(1.2)Sn(0.9)I(3)
$CsSnI_3$ S2	CsI and SnI_2 with additive NH_4Cl (ratio3:1)	1 M	DMF	Spin coating: 60s at 2000 rpm After 5s ODCB as antisolvent	10 min at 80 °C	5.02	5.76	4.42	Cs(1.2)Sn(0.9)I(3)
$CsSnI_3$ S3	CsI and SnI_2	0.5 M	DMF	Spin coating: 5s at 500 rpm, 40s at 1000 rpm and 50s at 5000 rpm	10 min at 100 °C	4.94	5.60	4.29	Cs(1.3)Sn(0.8)I(3)
$CsSnI_3$ S4 (UV-vis only)	CsI and SnI_2	0.5 M	DMSO	Spin coating: 5s at 500 rpm, 40s at 1000 rpm and 50 s at 5000 rpm	10 min at 80 °C	*Not measured*			
$MASnI_3$ S1	MAI and SnI_2	1 M	DMSO	Spin coating: 30s at 3000 rpm After 20s ODCB as antisolvent	10 min at 80 °C	4.70	5.38	4.04	C(1.2)N(0.9)Sn(0.8)I(3)
$MASnI_3$ S2	MAI and SnI_2	1 M	DMSO	Spin coating: 30s at 3000 rpm After 20s ODCB as antisolvent	30 s at 100 °C	4.72	5.36	4.00	C(1.3)N(1)Sn(0.8)I(3)
$MASnI_3$ S3	MAI and SnI_2 with additive NH_4Cl (1:0.84)	0.4 M	DMF	Spin coating: 30s at 3000 rpm	Not annealed	4.73	5.44	4.16	*Not measured*

Sample	Preparation (precursor, concentration, solvent, processing, annealing)					Wf [eV]	IE [eV]	EA [eV]	XPS stoichiometry
$MASnI_3$ S4 (UV-vis only)	MAI and SnI_2	1 M	DMSO	Spin coating: 50 s at 5000 rpm using ODCB antisolvent after 10 s.	10 min at 100 °C	*Not measured*			
$FASnI_3$ S1	FAI and SnI_2	1 M	DMSO	Spin coating: 30s at 3000 rpm After 20s ODCB as antisolvent	30 s at 100 °C	4.90	5.38	4.15	N(2.1)C(1)Sn(0.8)I(3)
$FASnI_3$ S2	FAI and SnI_2	1 M	DMSO	Spin coating: 5s at 500 rpm, 40s at 1000 rpm, and 50s at 5000 rpm After 33s of last step ODCB as antisolvent	10 min at 100 °C	4.77	5.41	4.15	N(2)C(1)Sn(0.9)I(3)
$FASnI_3$ S3	FAI and SnI_2	1 M	DMSO	Spin coating: 30s at 3000 rpm	10 min at 100 °C	4.88	5.24	4.06	N(2.5)C(1.3)Sn(0.65)I(3)
$FASnI_3$ S4 (UV-vis only)	FAI and SnI_2	1 M	DMSO	Spin coating: 5s at 500 rpm, 40s at 1000 rpm, and 50s at 5000 rpm After 20 s of last step Toluene as antisolvent	10 min at 80 °C	*Not measured*			
$CsSnBr_3$ S1	CsBr and $SnBr_2$	-	-	Co-evaporation at molar ratio 1 : 1	Not annealed	4.88	5.87	4.15	Cs(1.1)Sn(0.9)Br(3)
$CsSnBr_3$ S2	CsBr and $SnBr_2$	-	-	Co-evaporation at molar ratio 1.1 : 1 (CsBr:$SnBr_2$)	Not annealed	4.70	5.75	3.96	Cs(1.3)Sn(0.9)Br(3)
$CsSnBr_3$ S3	CsBr and $SnBr_2$	-	-	Co-evaporation at molar ratio 1 : 1	1 h at 60°C in vacuum	4.88	5.83	4.11	Cs(1.2)Sn(0.9)Br(3)
$MASnBr_3$ S1	MABr and $SnBr_2$	1 M	DMSO	Spin coating: 30s at 3000 rpm	10 min at 100 °C	4.60	5.71	3.40	C(1.7)N(1.3)Sn(1.1)Br(3)
$MASnBr_3$ S2	MABr and $SnBr_2$	1 M	DMSO	Spin coating: 30s at 3000 rpm	10 min at 100 °C	4.47	5.64	3.40	*Not measured*
$MASnBr_3$ S3	MABr and $SnBr_2$	1 M	DMSO	Spin coating: 30s at 3000 rpm	10 min at 100 °C	4.56	5.67	3.45	C(1.8)N(1.4)Sn(1)Br(3)
$FASnBr_3$ S1	FABr and $SnBr_2$	1 M	DMSO	Spin coating: 5s at 500 rpm, 40s at 1000 rpm, and 50s at 5000 rpm. After 33s of the last step ODCB as antisolvent	10 min at 100 °C	4.91	6.25	3.59	*Not measured*
$FASnBr_3$ S2	FABr and $SnBr_2$	0.5 M	DMSO	Spin coating: 5s at 500 rpm, 40s at 1000 rpm, and 50s at 5000 rpm. After 15s of last step ODCB as antisolvent	10 min at 100 °C	4.92	6.25	3.65	*Not measured*
$FASnBr_3$ S3	FABr and $SnBr_2$	1 M	DMSO	Spin coating: 5s at 500 rpm, 40s at 1000 rpm, and 50s at 5000 rpm. After 33s of last step ODCB as antisolvent	10 min at 100 °C	4.84	6.18	3.57	C(1.1)N(2.2)Sn(1)Br(3)
$CsSnCl_3$ S1	CsCl and $SnCl_2$	-	-	Co-evaporation at molar ratio 1 : 1	1 h at 80 °C in vacuum	4.97	6.42	3.52	Cs(1.1)Sn(1)Cl(3)
$CsSnCl_3$ S2	CsCl and $SnCl_2$	-	-	Co-evaporation at molar ratio 1 : 1.1 (CsI:$SnCl_2$)	Not annealed	5.06	6.48	3.43	Cs(1)Sn(1)Cl(3)
$CsSnCl_3$ S3	CsCl and $SnCl_2$	-	-	Co-evaporation at molar ratio 1 : 1	1 h at 60 °C in vacuum	5.00	6.41	3.47	Cs(1)Sn(1.1)Cl(3)

Sample	Preparation (precursor, concentration, solvent, processing, annealing)					Wf [eV]	IE [eV]	EA [eV]	XPS stoichiometry
$MASnCl_3$ S1	MACl and $SnCl_2$	1.92 M	DMSO	Spin coating: 30s at 4000 rpm After 5s CB as antisolvent	5 min at 110 °C	4.87	6.84	3.31	C(1.5)N(1)Sn(1)Cl(3)
$MASnCl_3$ S2	MACl and $SnCl_2$ with additive NH_4Cl (5:1)	1.5 M	DMF	Spin coating: 60s at 2000 rpm	10 min at 80 °C	4.67	6.98	3.53	C(1.7)N(1.4)Sn(1.1)Cl(3)
$MASnCl_3$ S3	MACl and $SnCl_2$	0.5 M	DMSO	Spin coating: 15s at 250 rpm and 25s at 1500 rpm	10 min at 100 °C	4.75	6.74	3.32	C(1.4)N(1)Sn(1.1)Cl(3)
$FASnCl_3$ S1	FACl and $SnCl_2$	0.5 M	DMSO	Soaking of 15 µl solution on sample for 30s, then spin coating 30s at 3000 rpm	10 min at 100 °C	4.86	7.36	3.78	C(1)N(2)Sn(1)Cl(3)
$FASnCl_3$ S2	FACl and $SnCl_2$	0.5 M	DMSO	Soaking of 15 µl solution on sample for 10s, then spin coating 30s at 3000 rpm	10 min at 100 °C	4.92	7.3	3.88	C(1)N(2)Sn(1)Cl(3)
$FASnCl_3$ S3	FACl and $SnCl_2$	0.5 M	DMSO	Spin coating: 15s at 250 rpm and 25s at 1500 rpm	10 min at 100 °C	4.57	7.05	3.24	*Not measured*
$CsPbI_3$ S1	CsI and PbI_2	0.5 M	DMF	Spin coating: 30s at 3000 rpm	10 min at 350 °C	4.66	6.25	4.47	Cs(1.2)Pb(0.8)I(3)
$CsPbI_3$ S2	CsI and PbI_2	1 M	DMF	Spin coating: 30s at 5000 rpm, After 5s CB as antisolvent	5 min 350 °C	4.72	6.32	4.55	Cs(1.2)Pb(0.9)I(3)
$CsPbI_3$ S3	CsI and PbI_2	1 M	DMF	Spin coating: 30s at 5000 rpm, After 5s CB as antisolvent	5 min 350 °C	4.73	6.19	4.39	*Not measured*
$MAPbI_3$ S1	MAI and PbI_2	1 M	DMF	Spin coating: 30s at 3000 rpm After 3s ODCB as antisolvent	1 h at 100 °C	4.41	5.90	4.31	C(1.1)N(1)Pb(0.9)I(3)
$MAPbI_3$ S2	MAI and PbI_2	1 M	DMF	Spin coating: 30s at 5000 rpm, After 5s CB as antisolvent	1 h at 100 °C	4.45	5.95	4.43	C(1)N(1)Pb(0.8)I(3)
$MAPbI_3$ S3	MAI and PbI_2	1 M	DMF	Spin coating: 30s at 5000 rpm, After 3s CB as antisolvent	1 h at 100 °C	4.41	5.94	4.33	C(1)N(0.9)Pb(1)I(3)
$FAPbI_3$ S1	FAI and PbI_2	1 M	DMF	Spin coating: 30s at 3000 rpm, After 4s CB as antisolvent	10 min at 140 °C	4.84	6.29	4.80	C(1)N(1.7)Pb(0.9)I(3)
$FAPbI_3$ S2	FAI and PbI_2	1 M	DMF	Spin coating: 30s at 5000 rpm, After 4s CB as antisolvent	20 min at 140 °C	5.23	6.30	4.83	*Not measured*
$FAPbI_3$ S3	FAI and PbI_2	1 M	DMF	Spin coating: 30s at 5000 rpm, After 4s CB as antisolvent	5 min at 140 °C	4.58	6.13	4.58	C(0.8)N(1.5)Pb(0.8)I(3)
$CsPbBr_3$ S1	CsBr and $PbBr_2$	-	-	Co-evaporation at molar ratio 1 : 1	Not annealed	5.32	6.5	4.16	Cs(1.1)Pb(0.9)Br(3)
$CsPbBr_3$ S2	CsBr and $PbBr_2$	-	-	Co-evaporation at molar ratio 1.1 : 1 (CsBr:$PbBr_2$)	Not annealed	5.20	6.44	4.17	Cs(1)Pb(1.1)Br(3)
$CsPbBr_3$ S3	CsBr and $PbBr_2$	0.2 M	DMSO	Spin coating: 5s at 500 rpm, 40s at 1000 rpm, 50s at 5000 rpm. After 20s of last step Toluene as antisolvent	10 min at 100 °C	5.06	6.56	4.17	Cs(1.1)Pb(0.7)Br(3)

Sample	Preparation (precursor, concentration, solvent, processing, annealing)					Wf [eV]	IE [eV]	EA [eV]	XPS stoichiometry
$MAPbBr_3$ S1	MABr and $PbBr_2$	1 M	DMSO	Spin coating: 5s at 500 rpm, 40s at 1000 rpm, 50s at 5000 rpm. After 6s of last step ODCB as antisolvent	10 min at 100 °C	5.33	6.59	4.26	*Not measured*
$MAPbBr_3$ S2	MABr and $PbBr_2$	1 M	DMSO	Spin coating: 5s at 500 rpm, 40s at 1000 rpm, 50s at 5000 rpm. After 6s of last step ODCB as antisolvent	10 min at 100 °C	5.27	6.60	4.21	C(1.7)N(1.3)Pb(1)Br(3)
$MAPbBr_3$ S3	$PbBr_2$ and MABr	0.5 M and 0.13 M	DMF and ml in IPA	First $PbBr_2$ spin coated 30s at 2000 rpm and pre-annealing at 80 °C, next 50 µl MABr solution on top and spin coated 30s at 2000 rpm	10 min at 80 °C	5.22	6.61	4.29	C(1.7)N(1.4)Pb(0.9)Br(3)
$MAPbBr_3$ S4 (XRD only)	MABr and $PbBr_2$	1 M	DMSO	Spin coating: at 3000 rpm for 30s	10min at 100C	*Not measured*			
$FAPbBr_3$ S1	FABr and $PbBr_2$	1 M	DMSO	Spin coating: 5s at 500 rpm, 40s at 1000 rpm, 50s at 5000 rpm. After 33s of last step ODCB as antisolvent	10 min at 100 °C	5.48	6.77	4.56	C(0.7)N(1.3)Pb(1)Br(3)
$FAPbBr_3$ S2	FABr and $PbBr_2$	0.5 M	DMSO	Spin coating: 5s at 500 rpm, 40s at 1000 rpm and 50s at 5000 rpm. After 10s of last step ODCB as antisolvent	10 min at 100 °C	5.39	6.67	4.43	C(1.1)N(1.7)Pb(1)Br(3)
$FAPbBr_3$ S3	FABr and $PbBr_2$	0.5 M	DMSO	Spin coating: 5s at 500 rpm, 40s at 1000 rpm, 50s at 5000 rpm. After 13 s of last step Toluene as antisolvent	10 min at 130 °C	5.41	6.67	4.54	C(1.3)N(2.1)Pb(1)Br(3
$CsPbCl_3$ S1	CsCl and $PbCl_2$	-	-	Co-evaporation at molar ratio 1 : 1	Not annealed	4.58	6.91	3.85	Cs(1)Pb(1)Cl(3)
$CsPbCl_3$ S2	CsCl and $PbCl_2$	-	-	Co-evaporation at molar ratio 1 : 1	1 h at 80 °C	4.3	6.76	3.77	Cs(1)Pb(0.9)Cl(3)
$CsPbCl_3$ S3	CsCl and $PbCl_2$	-	-	Co-evaporation at molar ratio 1 : 1	1 h at 80 °C	4.15	6.81	3.77	Cs(0.9)Pb(0.9)Cl(3)
$MAPbCl_3$ S1	MACl and $PbCl_2$	1.2 M	DMSO	Spin coating: 30s at 4000 rpm, after 20s CB was put on the spinning substrate	5 min at 110 °C	5.34	6.98	3.91	N(0.9)C(1.3)Pb(0.8)Cl(3)
$MAPbCl_3$ S2	$PbCl_2$ and MACl	1.4 M and 0.2 M	DMSO and IPA	First $PbCl_2$ spin coated 60s at 2000 rpm, next MABr solution on top, soaked for 10s then spun off at 2000 rpm for 60s	10 min at 100 °C	4.85	7.02	3.79	N(1.3)C(1.4)Pb(0.6)Cl(3)
$MAPbCl_3$ S3	MACl and $PbCl_2$	0.5 M	DMSO	Spin coating: 5s at 500 rpm, 40s at 1000 rpm, 50 s at 5000 rpm. After 13 s of last step Toluene as antisolvent	10 min at 100 °C	5.19	6.85	3.71	N(1)C(1.1)Pb(0.8)Cl(3)
$MAPbCl_3$ S4 (XRD only)	MACl and $PbCl_2$	0.5 M	DMSO	Spin coating: 60s at 2000 rpm	10 min at 100 °C	*Not measured*			

Sample	Preparation (precursor, concentration, solvent, processing, annealing)					Wf [eV]	IE [eV]	EA [eV]	XPS stoichiometry
$FAPbCl_3$ S1	FACl and $PbCl_2$	0.5 M	DMSO	Spin coating: 5s at 500 rpm, 40s at 1000 rpm, and 50s at 5000 rpm. After 13s of last step Toluene as antisolvent	10 min at 100 °C	5.03	6.97	4.05	*Not measured*
$FAPbCl_3$ S2	FACl and $PbCl_2$	0.5 M	DMSO	Spin coating: 5s at 500 rpm, 40s at 1000 rpm, 50s at 5000 rpm. After 15 s of last step ODCB as antisolvent	10 min at 100 °C	4.99	6.90	3.93	N(1.6)C(1)Pb(0.9)Cl(3)
$FAPbCl_3$ S3	FACl and $PbCl_2$	0.5 M	DMSO	Spin coating: 5s at 500 rpm, 40s at 100 rpm, 50s at 5000 rpm. After 15 s of last step ODCB as antisolvent	10 min at 100 °C	5.42	6.96	3.97	*Not measured*

3. Supplementary Notes and Figures

Comparisons between measured and DFT calculated densities of states

The following Supplementary Figure 6 shows all measurements used to extract IE and EA values listed in Table 1 of the main article; the different materials are divided in the subfigures a) to r). Here, the DFT data is shown at the top and the graphs below contain three different representative measurements, aligned to the theoretical DOS via the fitting procedure. Sample preparation is varied between these samples to avoid unintentional mistakes in film preparation; additional sample characterization and preparation protocols for each sample are reported in Supplementary Table 6 above as well as the Supplementary Notes 1 to 18 below.

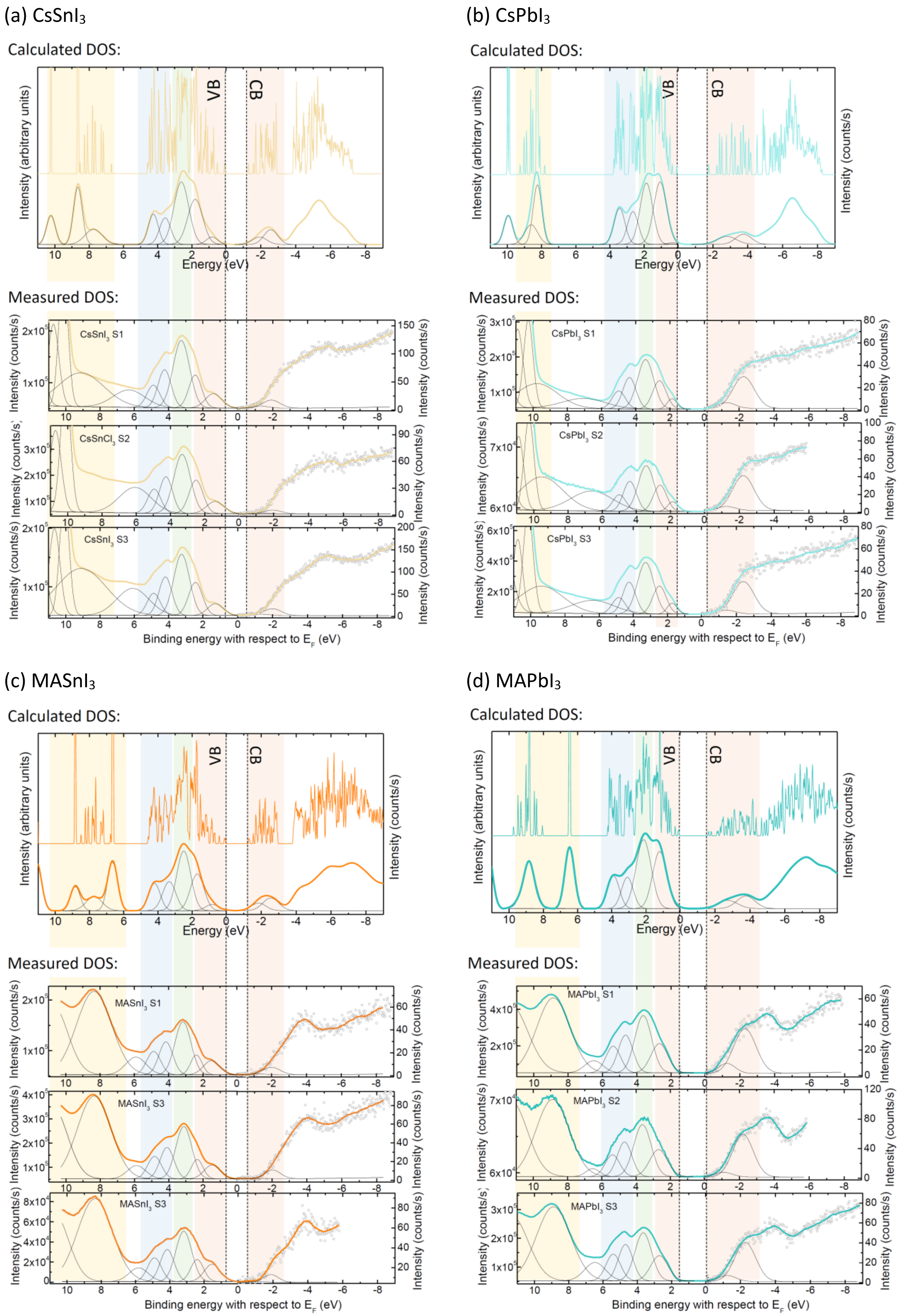
(a) CsSnI3
Calculated DOS:
VB
CB
Intensity (arbitrary units)
Energy (eV)
Measured DOS:
CsSnI3 S1
CsSnCl3 S2
CsSnI3 S3
Intensity (counts/s)
Binding energy with respect to EF (eV)
(b) CsPbI3
Calculated DOS:
VB
CB
Intensity (arbitrary units)
Intensity (counts/s)
Energy (eV)
Measured DOS:
CsPbI3 S1
CsPbI3 S2
CsPbI3 S3
Binding energy with respect to EF (eV)
(c) MASnI3
Calculated DOS:
VB
CB
Intensity (arbitrary units)
Intensity (counts/s)
Energy (eV)
Measured DOS:
MASnI3 S1
MASnI3 S3
MASnI3 S3
Binding energy with respect to EF (eV)
(d) MAPbI3
Calculated DOS:
VB
CB
Intensity (arbitrary units)
Intensity (counts/s)
Energy (eV)
Measured DOS:
MAPbI3 S1
MAPbI3 S2
MAPbI3 S3
Binding energy with respect to EF (eV)

(e) $FASnI_3$

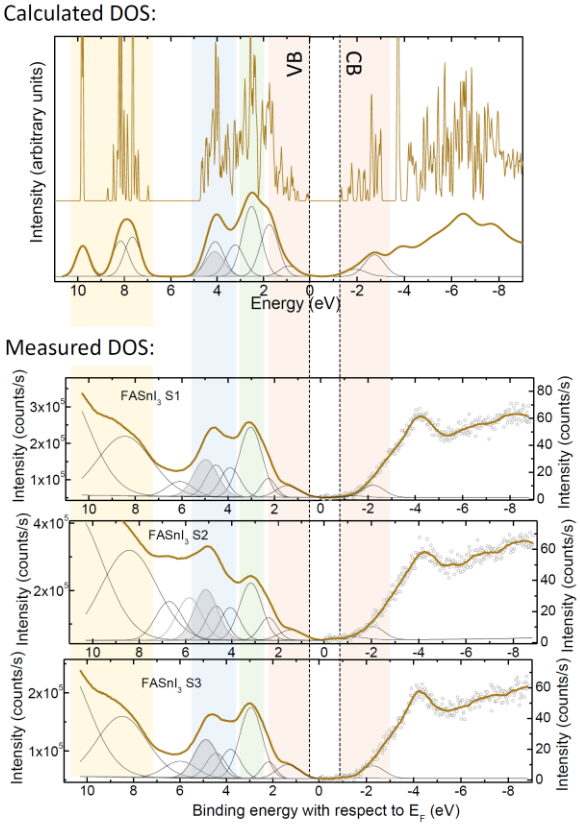

(f) $FAPbI_3$

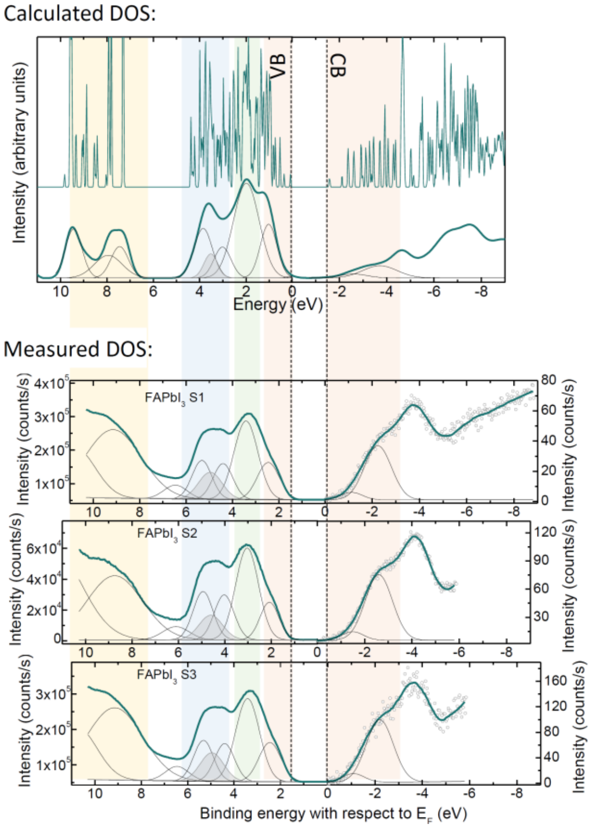

(g) $CsSnBr_3$

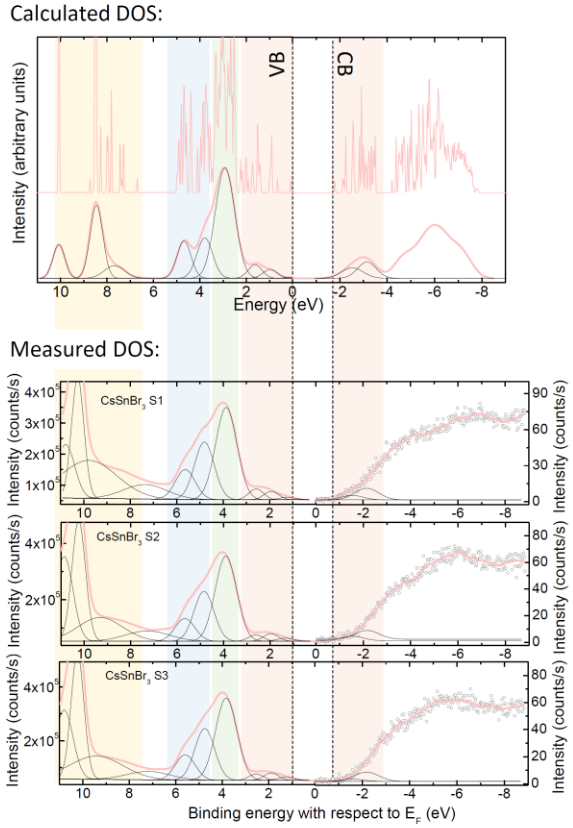

(h) $CsPbBr_3$

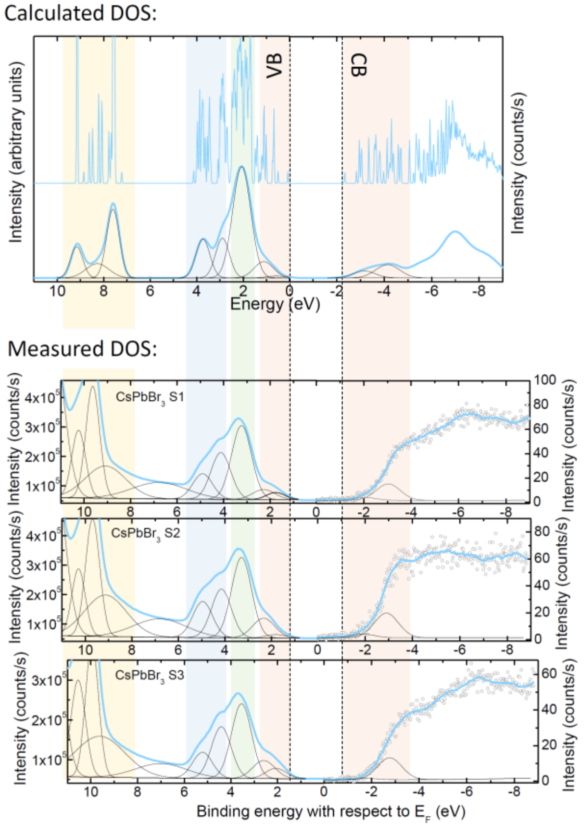

(i) $MASnBr_3$

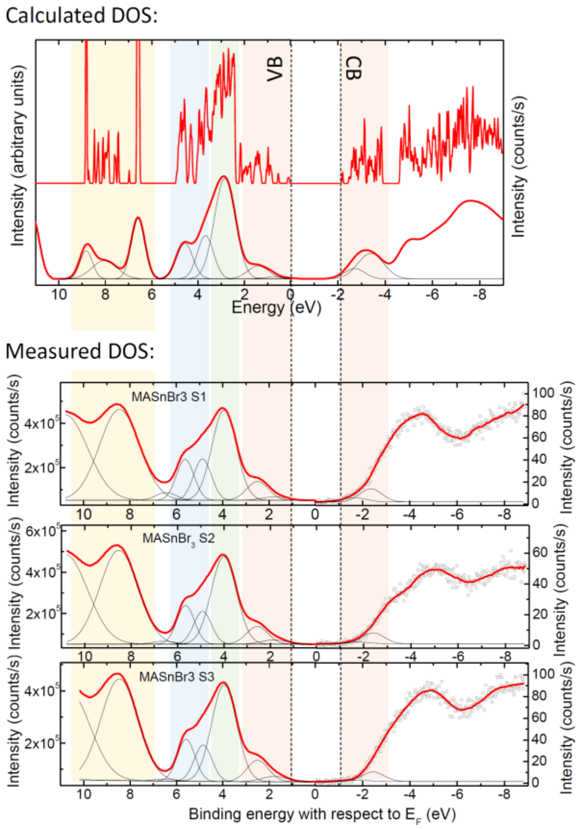

(j) $MAPbBr_3$

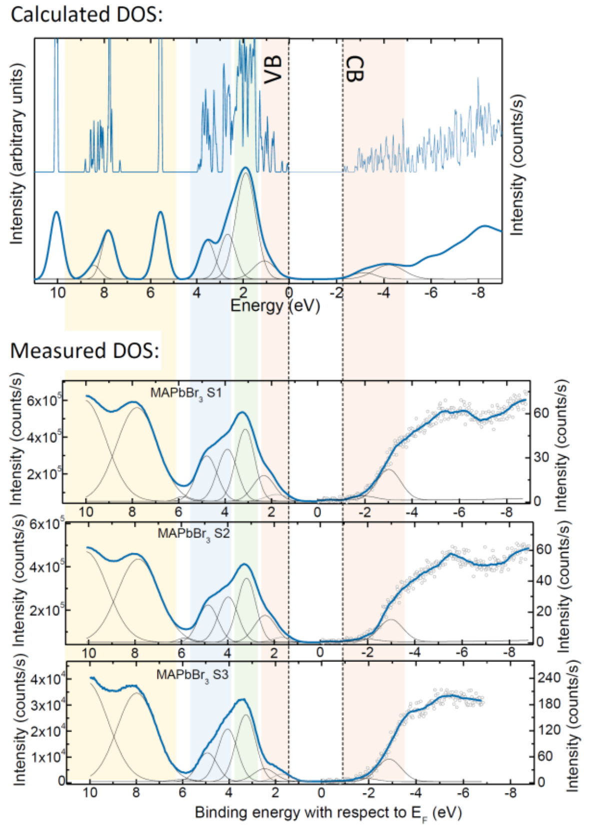

(k) $FASnBr_3$

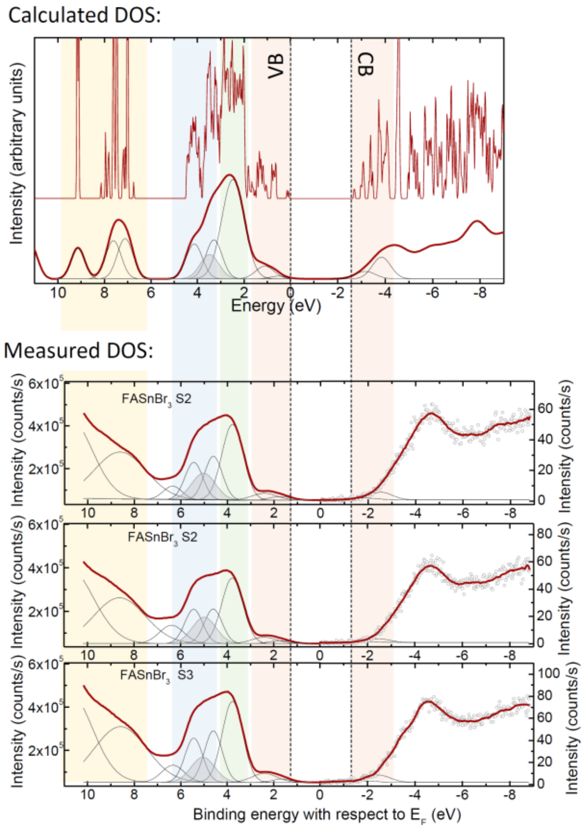

(l) $FAPbBr_3$

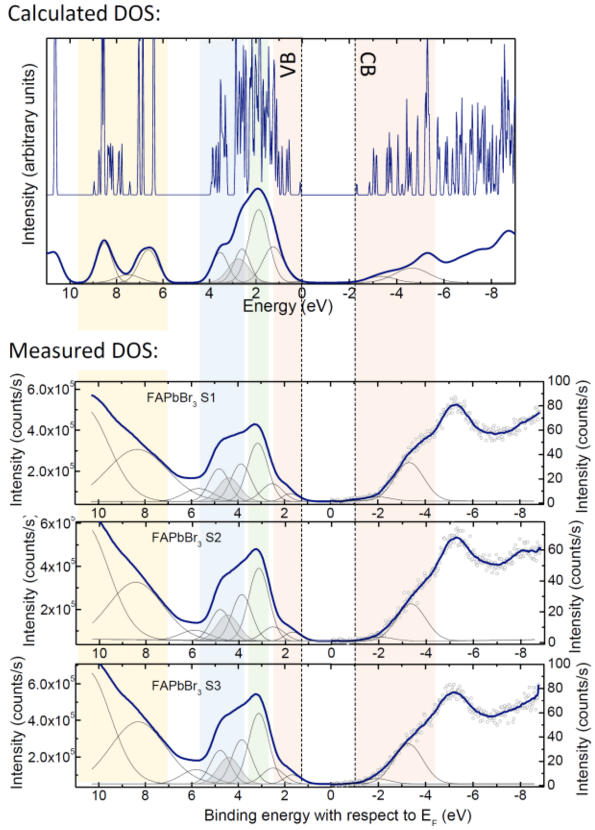

(m) $CsSnCl_3$

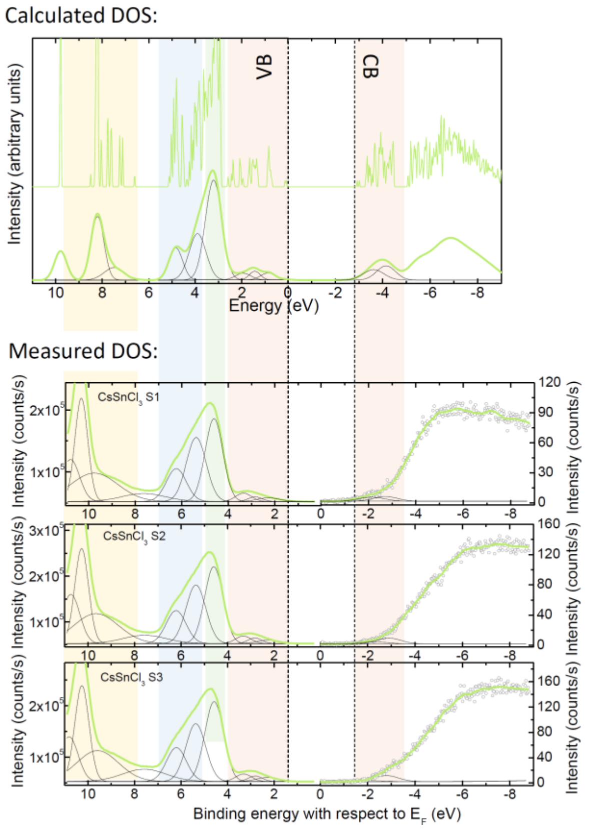

(n) $CsPbCl_3$

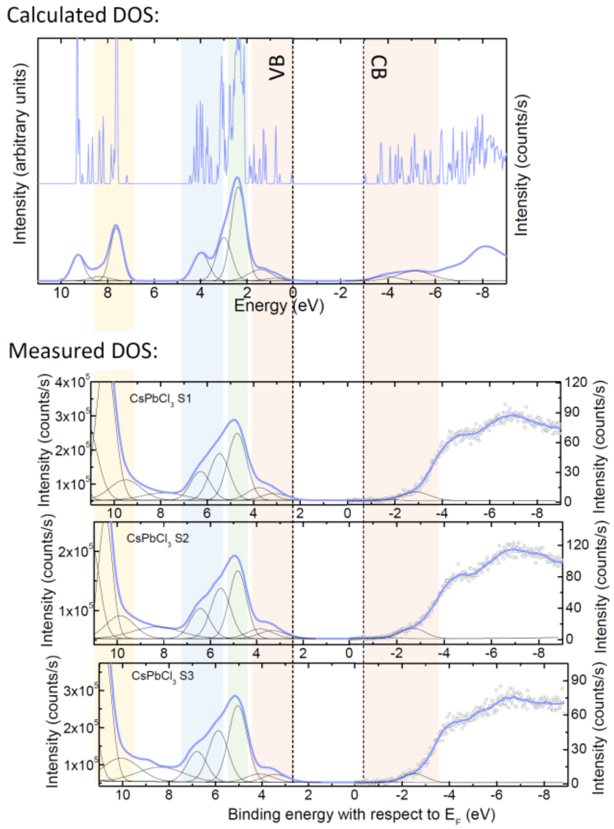

(o) $MASnCl_3$

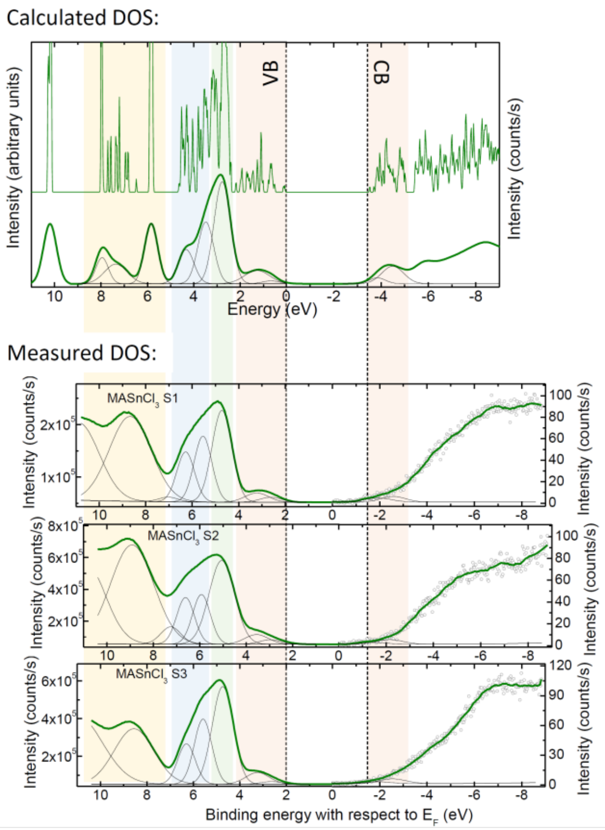

(p) $MAPbCl_3$

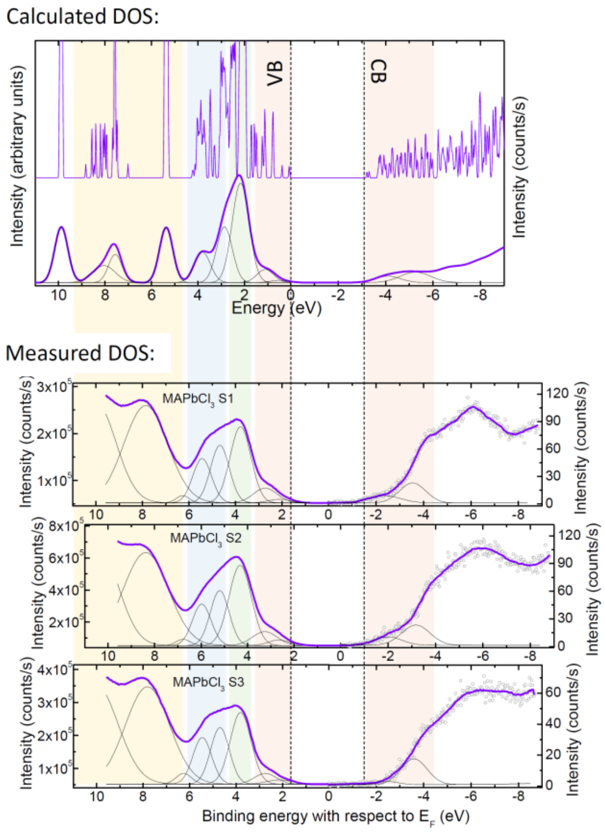

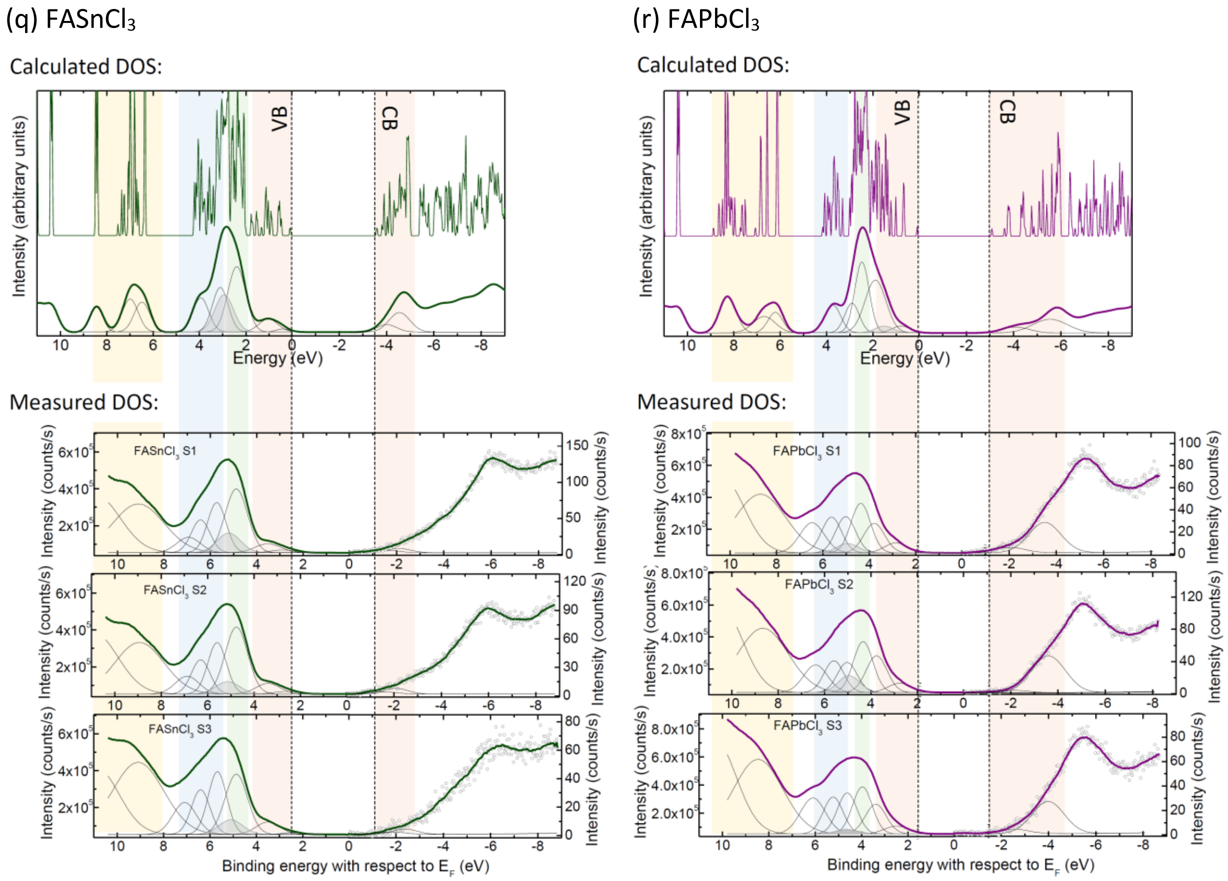

Supplementary Figure 6 | Comparisons between measured and DFT calculated density of states. All 18 perovskite systems are shown throughout the sub-figures a) to r). DFT and experimental spectra are aligned in order to extract the VB and CB onsets, which are indicated by dashed lines. Note that in each top graph, against DFT convention, positive values for VB and negative values for CB are used in analogy to the experimental plot. Shaded peaks show the presence of a FA derived feature, which is situated inside the main valence band. In the IPES measurements (negative binding energies) the open circles represent the measurement while the solid line is a smoothed curve through these data point. Sample numbers, such as “S1” etc., indicate the different preparation conditions, which can be found in Table 6.

Supplementary Material Datasets (XRD, SEM, XPS, UV-vis)

Supplementary Notes 1: $CsSnI_3$

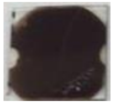

XRD measurement:

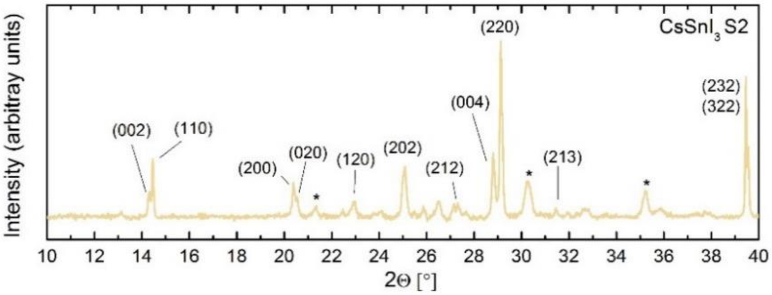

Orthorhombic crystal structure

a = 8.71 Å
b = 8.65 Å
c = 12.38 Å

*XRD measurement of $CsSnI_3$, sample 2; reflexes of the orthorhombic structures are marked and extracted lattice constants are displayed on the right. Reflexes marked with * come from the ITO substrate.*

SEM measurement:

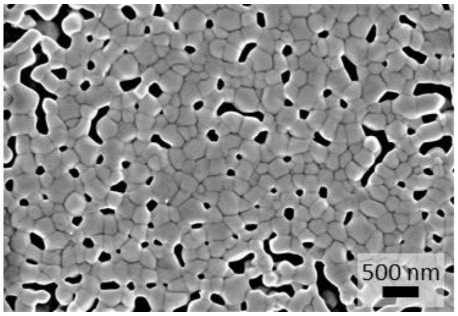

SEM image of $CsSnI_3$, sample 2.

Absorption measurement:

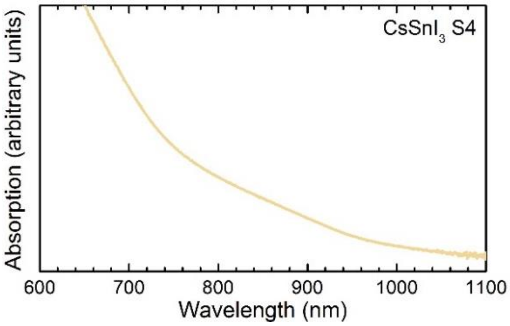

UV-vis measurement of $CsSnI_3$, sample 4.

XPS analysis:

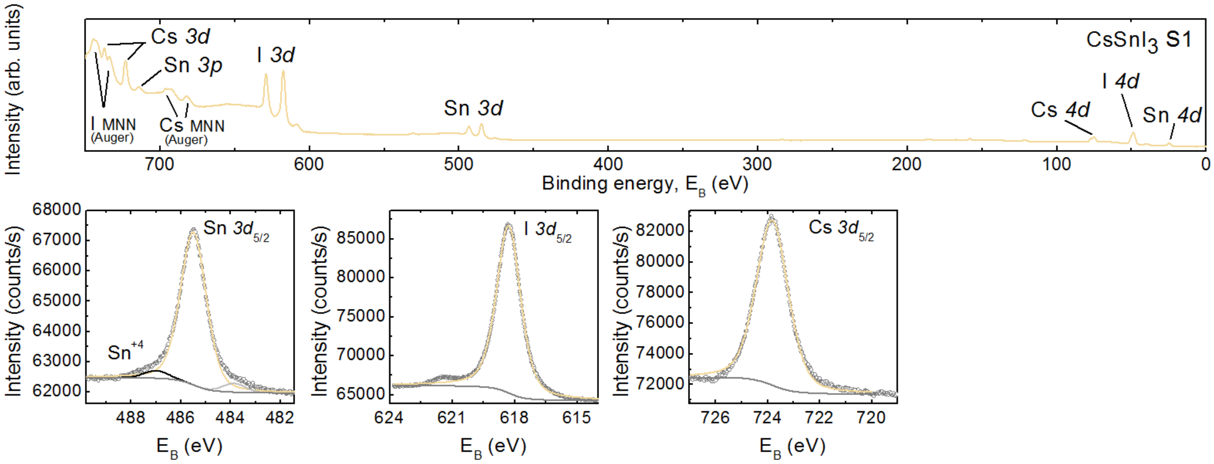

XPS measurement of $CsSnI_3$, sample 1, showing a survey spectrum on top as well as detailed scans of perovskite specific core level signals at the bottom; the solid lines are fits to the measurement (open circles).

Short discussion: In XRD, we find a large number of peaks, indicating an unordered film growth, which can be clearly associated with an orthorhombic crystal structure. Notable in the SEM image, it is usually difficult to form densely packed films for this material, probably due to limited solubility of CsI. XPS shows all expected features and oxidation states, but also a small shoulder for Sn at higher binding energies, likely originating from Sn^{+4} (approx. 6%). Note that the feature in the Sn signal at lower binding energy comes from an asymmetric peak shape and is not a metallic Sn feature (it is equally present in all Sn samples). UV-vis measurements yield an optical gap of 1.25 eV.

Supplementary Notes 2: $MASnI_3$

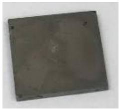

XRD measurement:

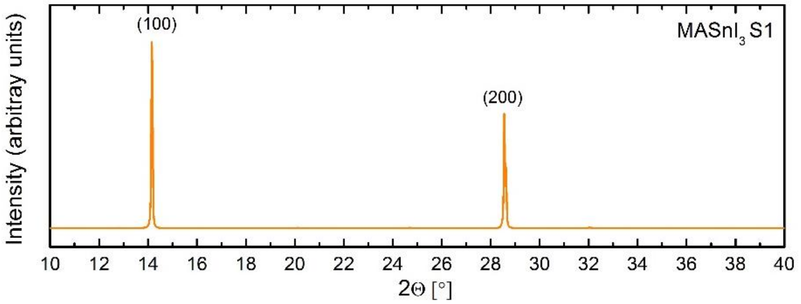

Cubic crystal structure

a = 6.25 Å

XRD measurement of $MASnI_3$, sample 1; reflexes of the cubic structure are marked and the extracted lattice constant is displayed on the right.

SEM:

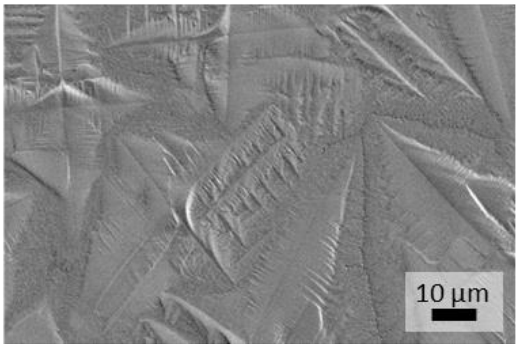

SEM image of $MASnI_3$, sample 1.

Absorption:

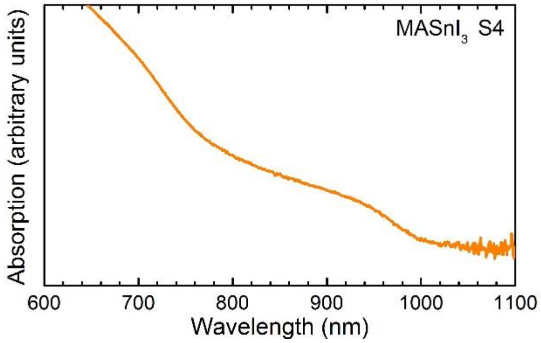

UV-vis measurement of $MASnI_3$, sample 4.

XPS analysis:

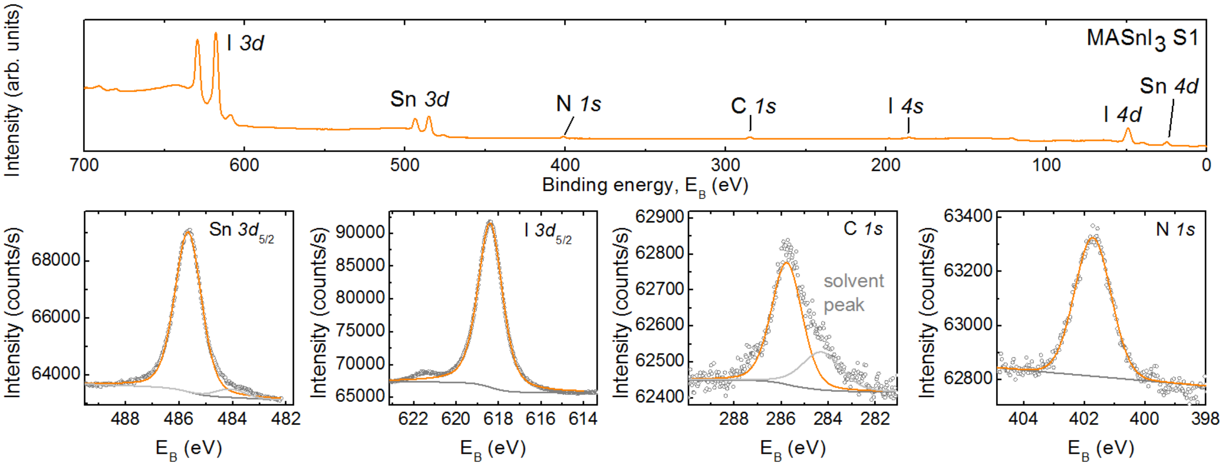

XPS measurement of $MASnI_3$, sample 1, showing a survey spectrum on top as well as detailed scans of perovskite specific core level signals at the bottom; the solid lines are fits to the measurement (open circles).

Short discussion: XRD of $MASnI_3$ consistently shows highly ordered films, with only (h00) reflexes, indicating a preferred crystallite orientation. The low number of reflexes does not allow to unambiguously tell the crystal structure, but from powder diffraction studies in literature it is known to be cubic [13]. This material forms large intercalated crystals, usually in a “leaf” like structure as seen in the SEM image. XPS shows all expected features and oxidation states with some additional more neutral carbon species (likely solvent remaining in the film). Note that the feature in the Sn signal at lower binding energy comes from an asymmetric peak shape and is not a metallic Sn feature (it is equally present in all Sn samples). UV-vis measurements yield an optical gap of 1.24 eV.

Supplementary Notes 3: $FASnI_3$

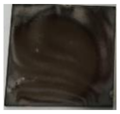

XRD measurement:

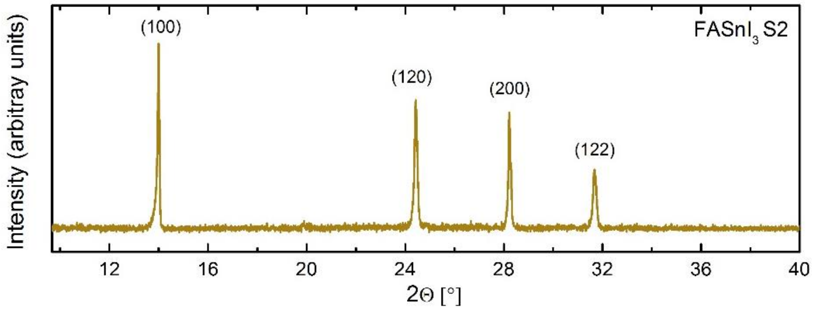

XRD measurement of $FASnI_3$, sample S2; reflexes of the tetragonal structure are marked and extracted lattice constants are displayed on the right.

Tetragonal crystal structure

a = 6.32 Å
b = c = 8.93 Å

SEM:

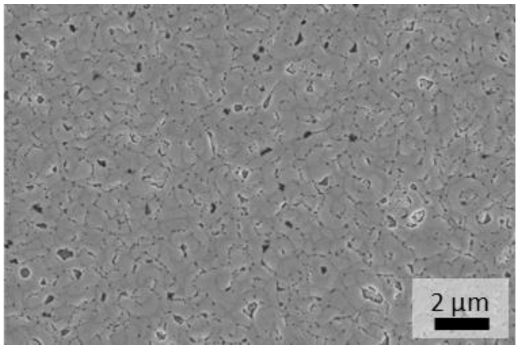

SEM image of $FASnI_3$, sample 1.

Absorption:

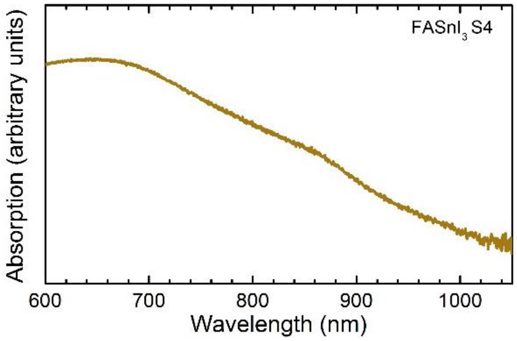

UV-vis measurement of $FASnI_3$, sample 4.

XPS analysis:

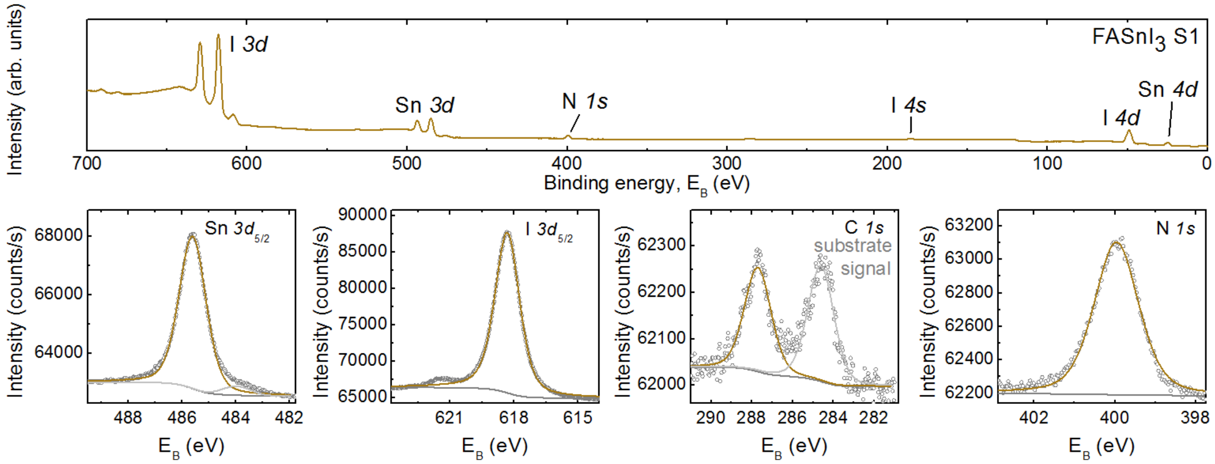

XPS measurement of $FASnI_3$, sample 1, showing a survey spectrum on top as well as detailed scans of perovskite specific core level signals at the bottom; the solid lines are fits to the measurement (open circles).

Short discussion: XRD of $FASnI_3$ consistently shows all reflexes associated with a cubic structure indicating that no preferred crystal orientation is present. It is challenging to form dense films, usually "leaf" like structures similar to the $MASnI_3$ case are observed (not shown) or smoother layers with some pinholes, as shown in the SEM image. XPS shows all expected features and oxidation states, the additional carbon peak likely originates from the substrate that is visible through pin holes. Note that the feature in the Sn signal at lower binding energy comes from an asymmetric peak shape and is not a metallic Sn feature (it is equally present in all Sn samples). UV-vis measurements yield an optical gap of 1.24 eV, the onset is weak since the detector is not working efficiently any more in this energy range.

Supplementary Notes 4: $CsSnBr_3$

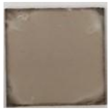

XRD measurement:

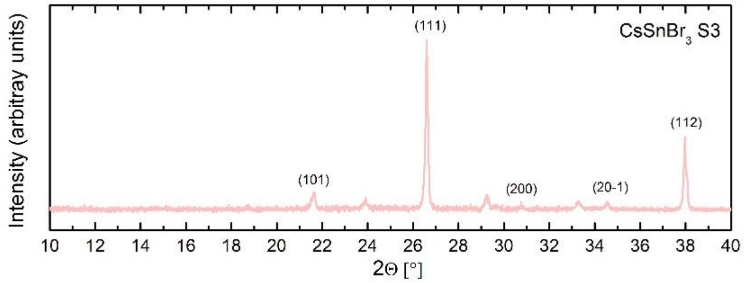

Cubic crystal structure

a = 5.81 Å

XRD measurement of $CsSnBr_3$, sample 3; reflexes of the cubic structure are marked and the extracted lattice constant is displayed on the right.

SEM:

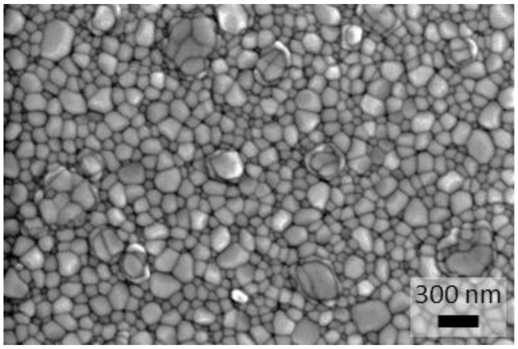

SEM image of $CsSnBr_3$, sample 1.

Absorption:

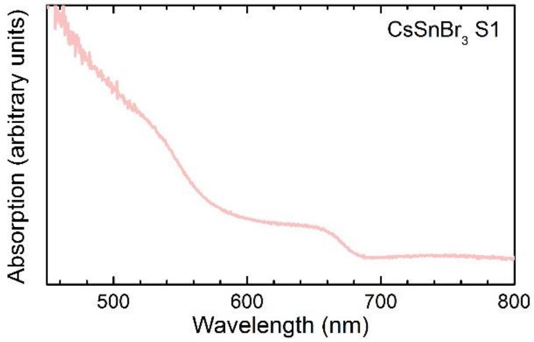

UV-vis measurement of $CsSnBr_3$, sample 1.

XPS analysis:

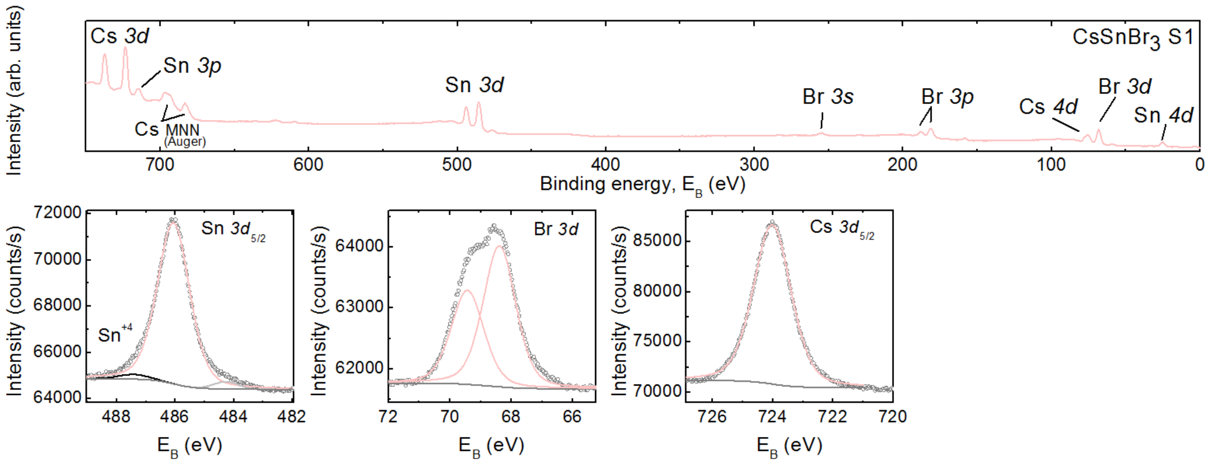

XPS measurement of $CsSnBr_3$, sample 1, showing a survey spectrum on top as well as detailed scans of perovskite specific core level signals at the bottom; the solid lines are fits to the measurement (open circles).

Short discussion: In XRD measurements of evaporated $CsSnBr_3$, such as the one shown above, the (h00) peak is missing, indicating preferred film grown along the (111) direction for this cubic system, which is in contrast to other perovskite materials. Samples prepared by solution processing usually show no preferred orientation, but always a residual CsBr feature appears due to issues with solubility; no dense films can be formed. Therefore, only evaporated layers are presented in this paper, which form dense crystalline films, as seen in the SEM image. XPS shows all expected features and oxidation states, but also a small shoulder at higher binding energies for Sn, likely originating from Sn^{+4} (less than 3% signal). Note that the feature in the Sn signal at lower binding energy comes from an asymmetric peak shape and is not a metallic Sn feature (it is equally present in all Sn samples). UV-vis yields an optical gap of 1.81 eV.

Supplementary Notes 5: $MASnBr_3$

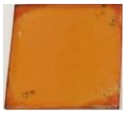

XRD measurement:

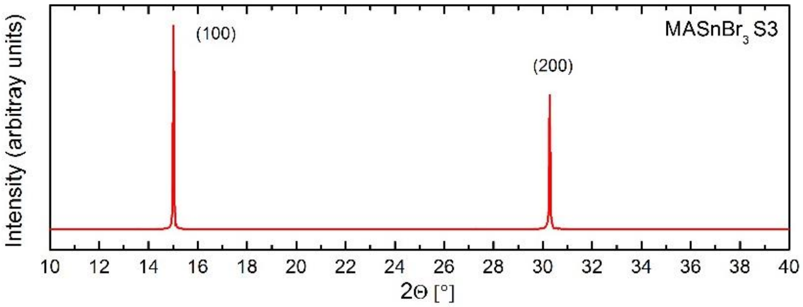

Cubic crystal structure

a = 5.90 Å

XRD measurement of $MASnBr_3$, sample 3; reflexes of the cubic structure are marked and the extracted lattice constant is displayed on the right.

SEM:

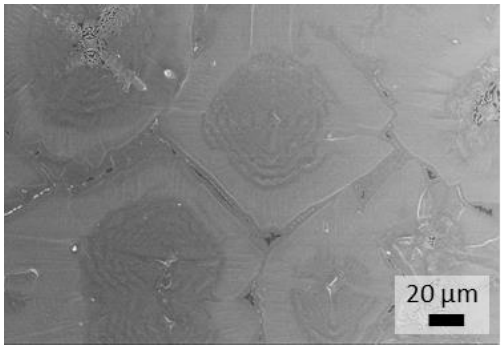

SEM image of $MASnBr_3$, sample 3.

Absorption:

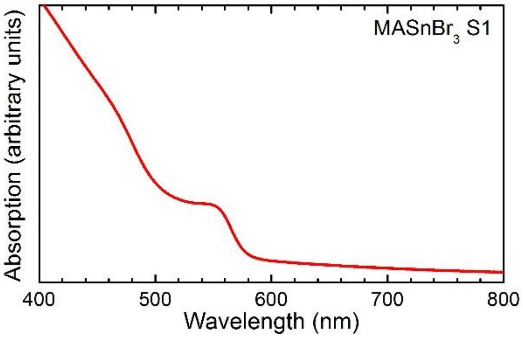

UV-vis measurement of $MASnBr_3$, sample 1.

XPS analysis:

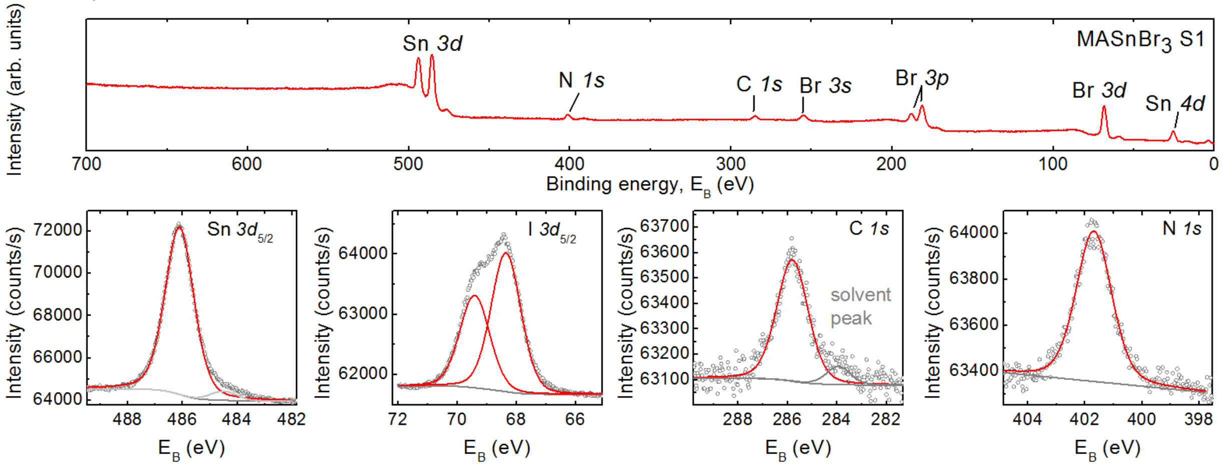

XPS measurement of $MASnBr_3$, sample 1, showing a survey spectrum on top as well as detailed scans of perovskite specific core level signals at the bottom; the solid lines are fits to the measurement (open circles).

Short discussion: XRD of $MASnBr_3$ consistently shows highly ordered films, with only (h00) reflexes, indicating a preferred crystal orientation. The low number of reflexes does not allow to unambiguously identify the crystal structure, but from powder diffraction studies in literature it is known to be cubic [14]. Like many of the tin based systems, this material forms large domains as seen in the SEM image. XPS shows all expected features and oxidation states with some additional more neutral carbon species (likely solvent remaining in the film). Note that the feature in the Sn signal at lower binding energy comes from an asymmetric peak shape and is not a metallic Sn feature (it is equally present in all Sn samples). UV-vis yields an optical gap of 2.15 eV.

Supplementary Notes 6: $FASnBr_3$

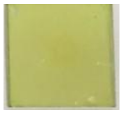

XRD measurement:

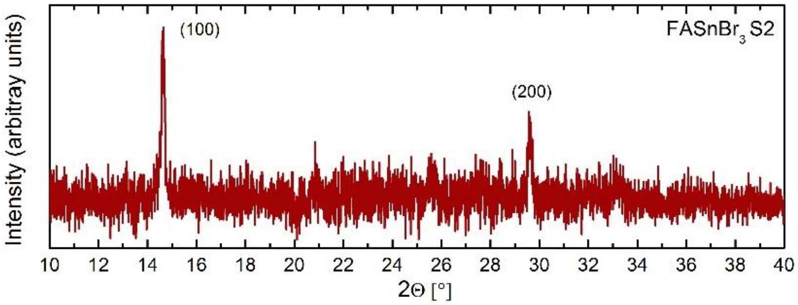

Cubic crystal structure

a = 6.05 Å

XRD measurement of $FASnBr_3$, sample 2; reflexes of the cubic structure are marked and the extracted lattice constant is displayed on the right.

SEM:

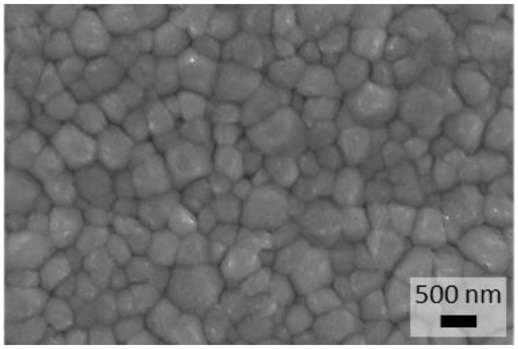

SEM image of $FASnBr_3$, sample 3.

Absorption:

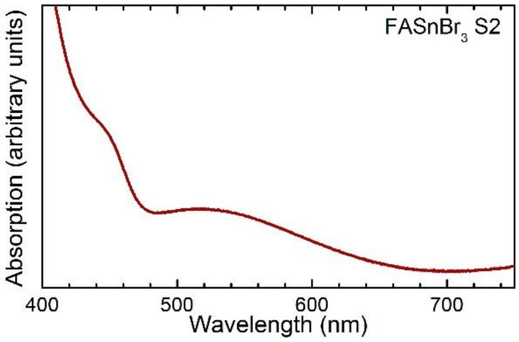

UV-vis measurement of $FASnBr_3$, sample 2.

XPS analysis:

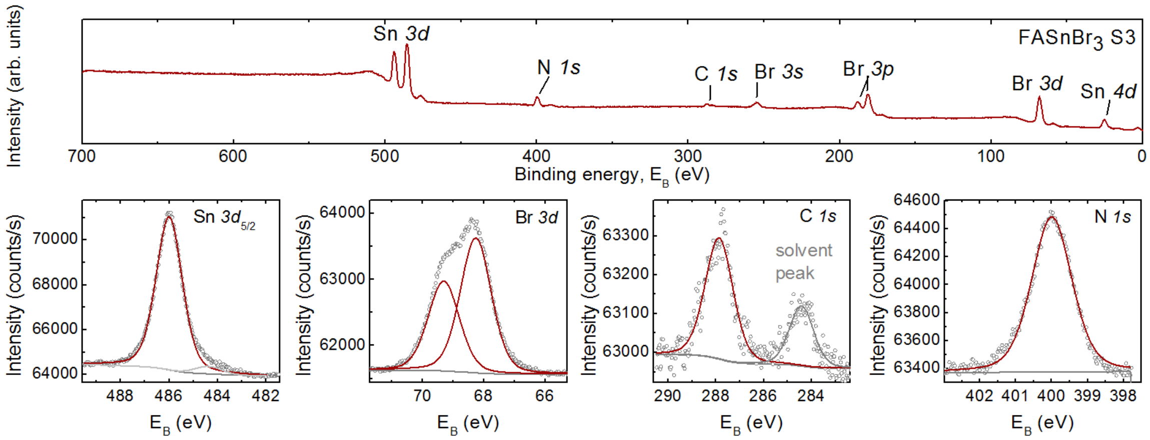

XPS measurement of $FASnBr_3$, sample 3, showing a survey spectrum on top as well as detailed scans of perovskite specific core level signals at the bottom; the solid lines are fits to the measurement (open circles).

Short discussion: In XRD we are only able to record rather noisy spectra, indicating that the crystallinity is not very good, even though SEM measurements show nicely formed films with typically close-packed crystallites. There seems to be a preferred orientation, but from the few reflexes it is not possible to unambiguously determine the crystal structure; from literature we can infer that it should be cubic [15]. This low crystallinity is consistent with the discussion in the main article, where we find that lattice must be very distorted due to the size difference between the small Sn/Br and the large FA, leading to the destabilization of the valance band. XPS shows all expected features and oxidation states with some additional more neutral carbon species (likely solvent remaining in the film). Note that the feature in the Sn signal at lower binding energy comes from an asymmetric peak shape and is not a metallic Sn feature (it is equally present in all Sn samples). UV-vis yields an optical gap of 2.63 eV.

Supplementary Notes 7: $CsSnCl_3$

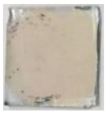

XRD measurement:

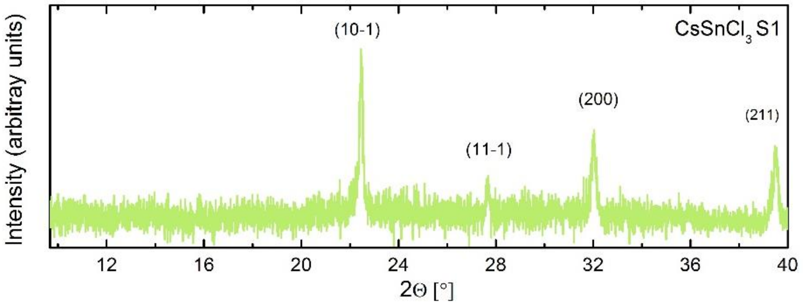

Cubic crystal structure

a = 5.59 Å

XRD measurement of $CsSnCl_3$, sample 1; reflexes of the cubic structure are marked and the extracted lattice constant is displayed on the right.

SEM:

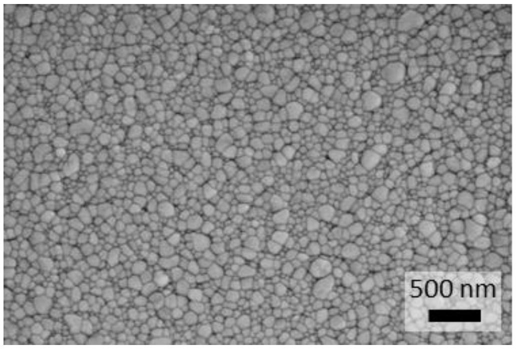

SEM image of $CsSnCl_3$, sample 3.

Absorption:

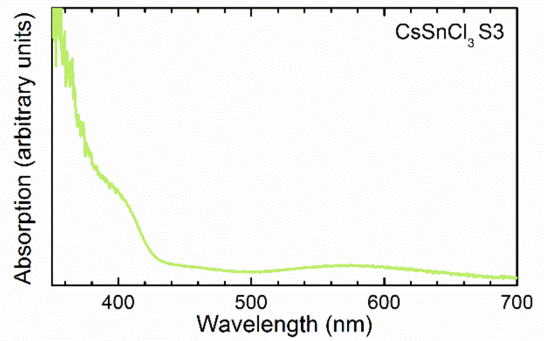

UV-vis measurement of $CsSnCl_3$, sample 3.

XPS analysis:

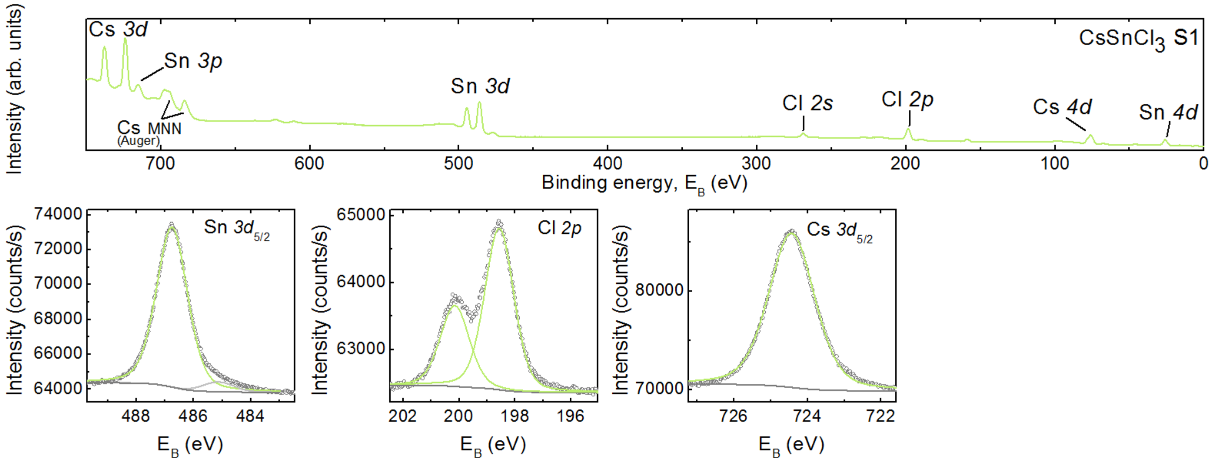

XPS measurement of $CsSnI_3$, sample 1, showing a survey spectrum on top as well as detailed scans of perovskite specific core level signals at the bottom; the solid lines are fits to the measurement (open circles).

Short discussion: All samples were evaporated, since by solution processing no perovskite could be formed (limited solubility of CsCl). Generally, this material does not show good crystallinity in XRD and similar to the case of $CsSnBr_3$ the (100) reflex is missing, indicating a preferred orientation. The few reflexes indicate a cubic structure, which is in agreement with high temperature measurements reported in literature [16]. SEM images show densely packed crystallites, which is typical for vapor deposition. XPS shows all expected features and oxidation states. Note that the feature in the Sn signal at lower binding energy comes from an asymmetric peak shape and is not a metallic Sn feature (it is equally present in all Sn samples). UV-vis measurements yield an optical gap of 2.88 eV.

Supplementary Notes 8: $MASnCl_3$

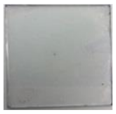

XRD measurement:

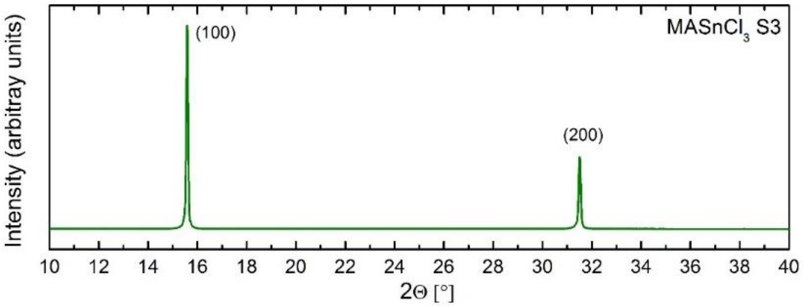

XRD measurement of $MASnCl_3$, sample 3; detectable reflexes are marked.

Monoclinic crystal structure

lattice constants cannot be extracted from this data set.

SEM:

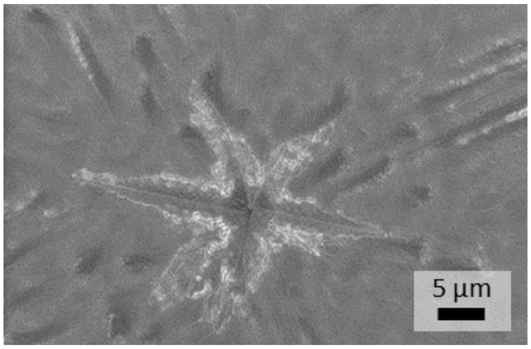

SEM image of $MASnCl_3$, sample 3.

Absorption:

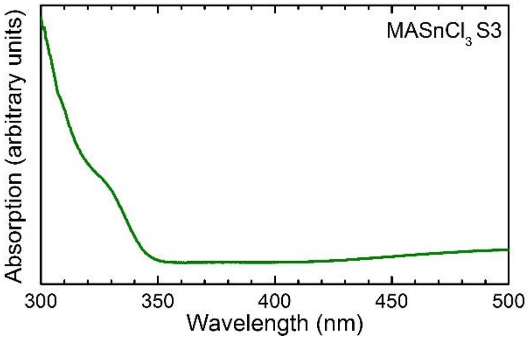

UV-vis measurement of $MASnCl_3$, sample 3.

XPS analysis:

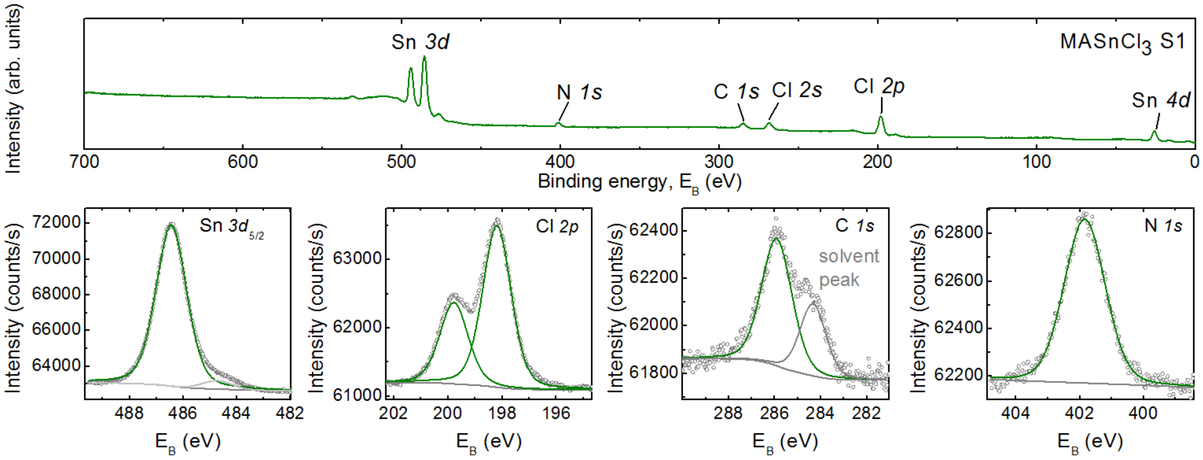

XPS measurement of $MASnCl_3$, sample 1, showing a survey spectrum on top as well as detailed scans of perovskite specific core level signals at the bottom; the solid lines are fits to the measurement (open circles).

Short discussion: The XRD spectrum indicates a cubic structure however one cannot be certain due to the low number of diffraction peaks. From literature it is known that this material is monocline at room temperature [17]; from our data set the exact lattice constants are therefore not extractable as we only see two reflexes. The before mentioned paper reports a =5.69 Å, b = 8.231 Å, c = 7.94 Å, β = 93.2° and their positions of the (100) and (200) reflexes are in good agreement with the current work. SEM measurements show large domains with “leaf” like structure and dense film formation. XPS shows all expected features and oxidation states with some additional more neutral carbon species (likely solvent remaining in the film). Note that the feature in the Sn signal at lower binding energy comes from an asymmetric peak shape and is not a metallic Sn feature (it is equally present in all Sn samples). UV-vis yields an optical gap of 3.58 eV.

Supplementary Notes 9: $FASnCl_3$

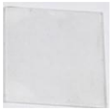

XRD measurement:

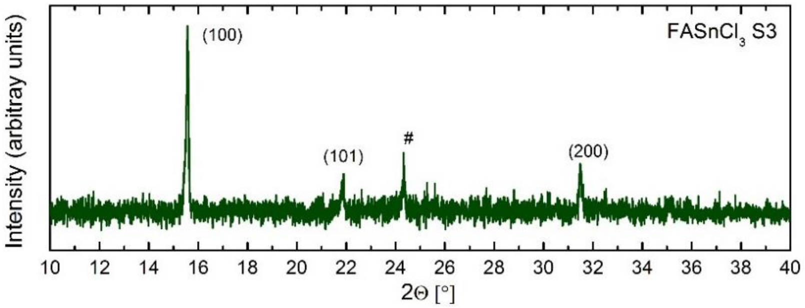

Cubic crystal structure (assumed)

a = 5.69 Å

XRD measurement of $FASnCl_3$, sample S3; reflexes of the cubic structure are marked and the extracted lattice constant is displayed on the right.

SEM:

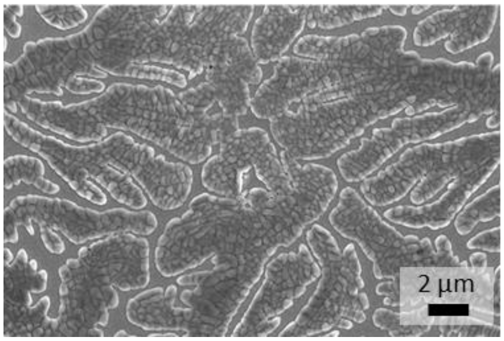

SEM image of $FASnCl_3$, sample 2.

Absorption:

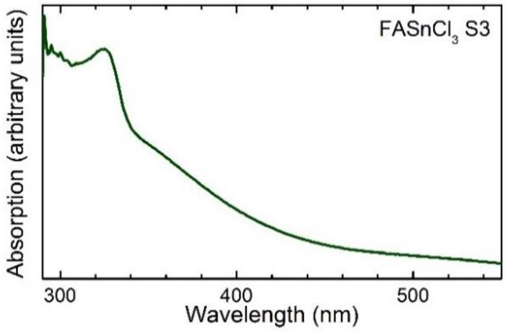

UV-vis measurement of $FASnCl_3$, sample 3.

XPS analysis:

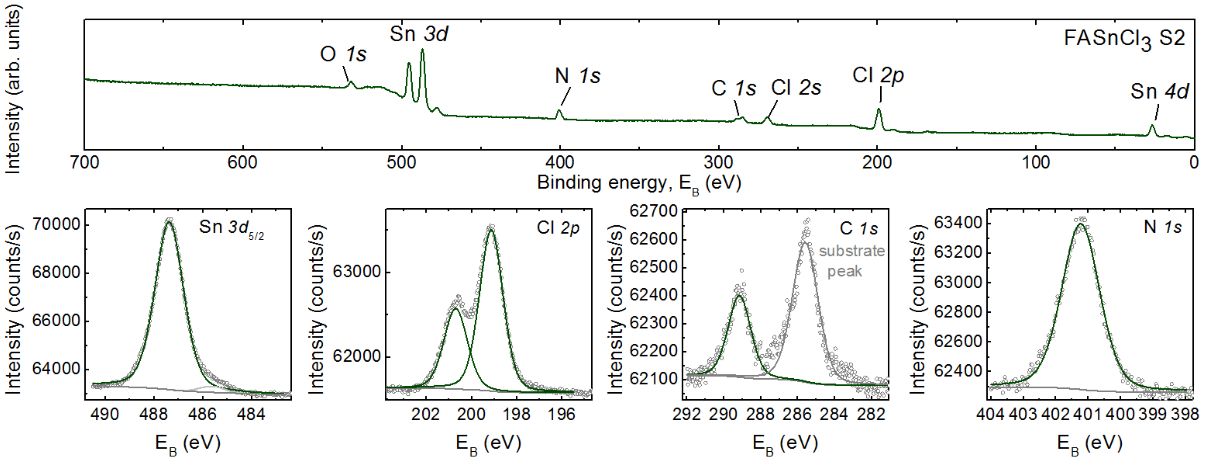

XPS measurement of $FASnCl_3$, sample 2, showing a survey spectrum on top as well as detailed scans of perovskite specific core level signals at the bottom; the solid lines are fits to the measurement (open circles).

Short discussion: The XRD spectrum only shows a low number of diffraction peaks indicating high order, however, it is unclear where the reflection marked as (#) originates from. We assume the crystal structure to be cubic, even though this cannot be unambiguously determined from a thin film measurement; in literature no thin film or powder XRD data have been reported. SEM images show densely packed crystallites even though the substrate is not fully covered. This is notable in the XPS measurements as well, where a significant additional carbon peak is dominated by a signal from the underlying PEDOT:PSS. All other elements show the expected oxidation states. Note that the feature in the Sn signal at lower binding energy comes from an asymmetric peak shape and is not a metallic Sn feature (it is equally present in all Sn samples). UV-vis yields an optical gap of 3.51 eV.

Supplementary Notes 10: $CsPbI_3$

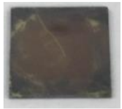

XRD measurement:

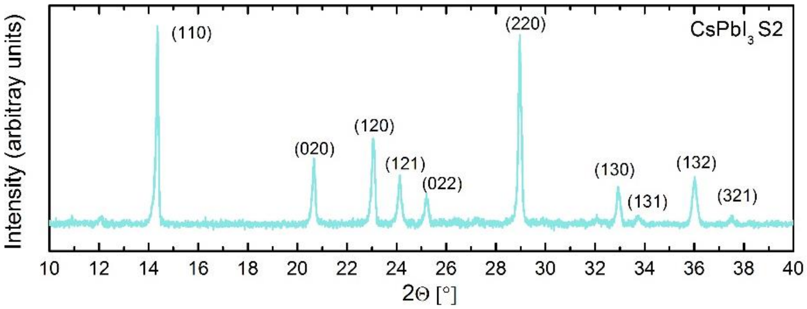

Orthorhombic crystal structure

a = 8.77 Å
b = 8.59 Å
c = 12.41 Å

XRD measurement of $CsPbI_3$, sample S2; reflexes of the orthorhombic structure are marked and the extracted lattice constants are displayed on the right.

SEM:

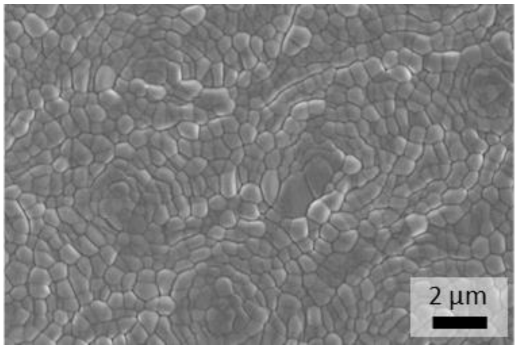

SEM image of $CsPbI_3$, sample 1.

Absorption:

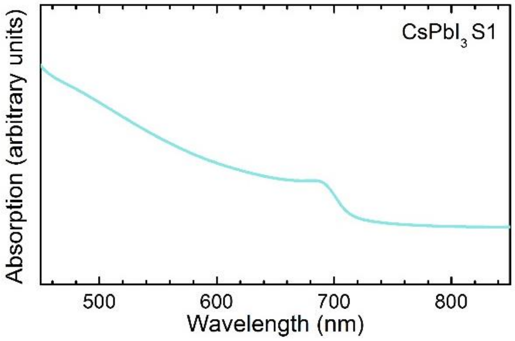

UV-vis measurement of $CsPbI_3$, sample 2.

XPS analysis:

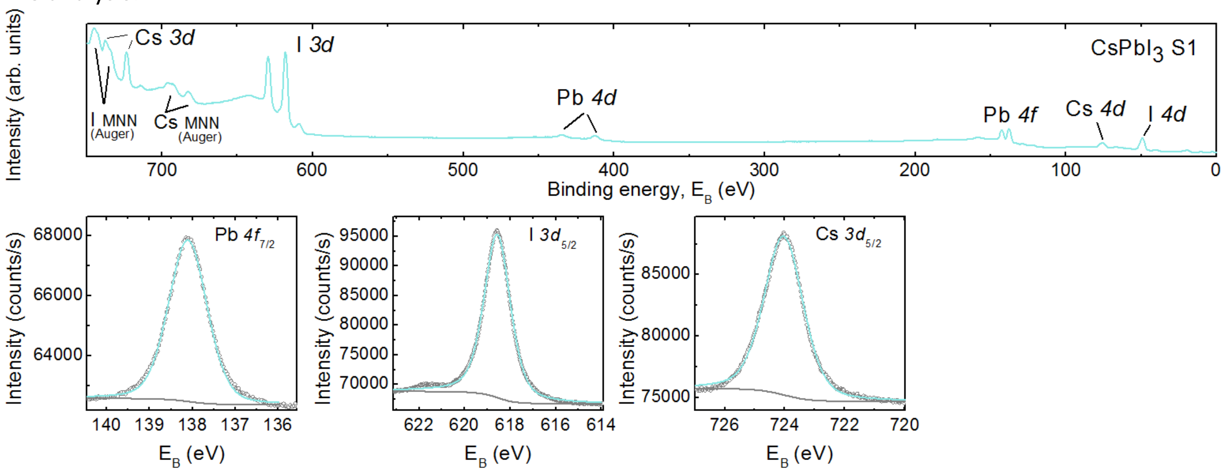

XPS measurement of $CsPbI_3$, sample 1, showing a survey spectrum on top as well as detailed scans of perovskite specific core level signals at the bottom; the solid lines are fits to the measurement (open circles).

Short discussion: $CsPbI_3$ is commonly reported to be cubic at room temperature, which however does not agree with our spectra. Recently, Sutton et al [18] published an orthorhombic structure, which is in excellent agreement with the XRD spectrum shown above and was used to identify the reflexes here. SEM images show densely packed films, quite similar to what is found for $MAPbI_3$. XPS peak analysis shows only the expected features and oxidation states. UV-vis yields an optical gap of 1.73 eV.

Supplementary Notes 11: $MAPbI_3$

XRD measurement:

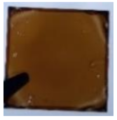

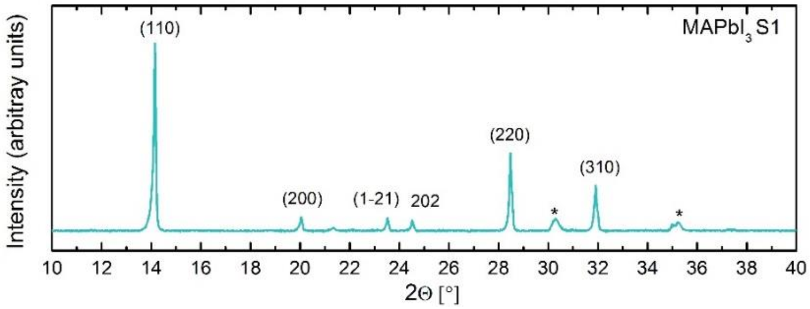

Tetragonal crystal structure

a = b = 8.88 Å
c = 12.68 Å

*XRD measurement of $MAPbI_3$, sample 1; reflexes of the tetragonal structure are marked and extracted lattice constants are displayed on the right. Reflexes marked with * come from underlying ITO substrate.*

SEM:

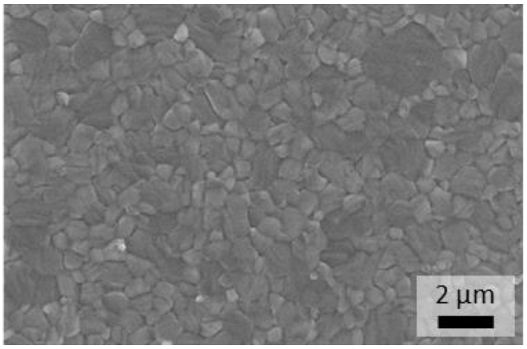

SEM image of $MAPbI_3$, sample 2.

Absorption:

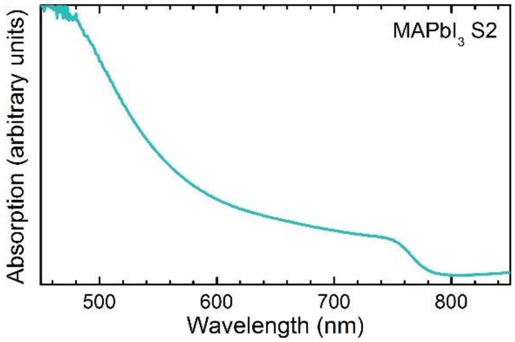

UV-vis measurement of $MAPbI_3$, sample 2.

XPS analysis:

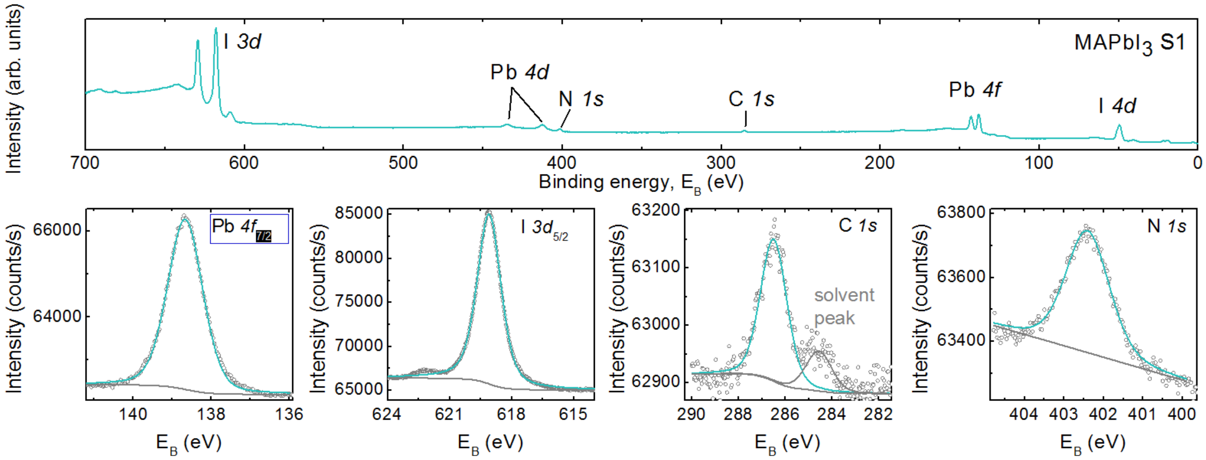

XPS measurement of $MAPbI_3$, sample 1, showing a survey spectrum on top as well as detailed scans of perovskite specific core level signals at the bottom; the solid lines are fits to the measurement (open circles).

Short discussion: XRD measurements show the well-known tetragonal crystal structure, usually with a preferential growth in the (110) direction. Peaks with asterisk (*) originate from underlying ITO substrate. SEM measurements show the typical close packed crystallites and XPS show all expected peaks and oxidation states with some additional more neutral carbon species (likely solvent remaining in the film). UV-vis yields an optical gap of 1.59 eV.

Supplementary Notes 12: $FAPbI_3$

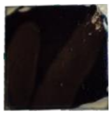

XRD measurement:

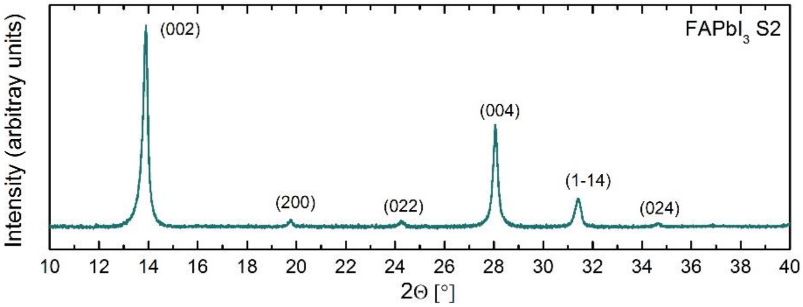

Tetragonal crystal structure

a = b = 8.99 Å
c = 12.75 Å

XRD measurement of $FAPbI_3$, sample S2; reflexes of the tetragonal structure are marked and extracted lattice constants are displayed on the right.

SEM:

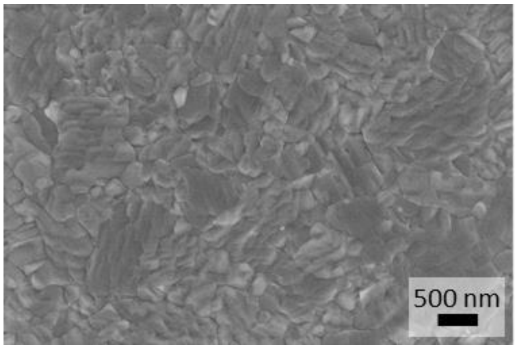

SEM image of $FAPbI_3$, sample 1.

Absorption:

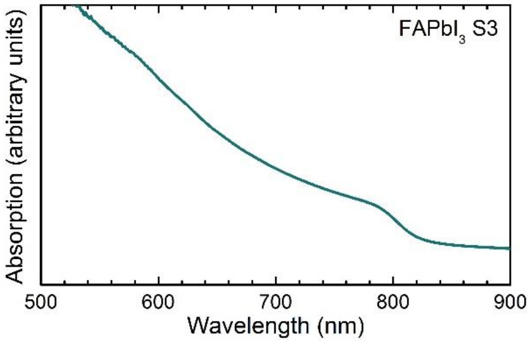

UV-vis measurement of $FAPbI_3$, sample 3.

XPS analysis:

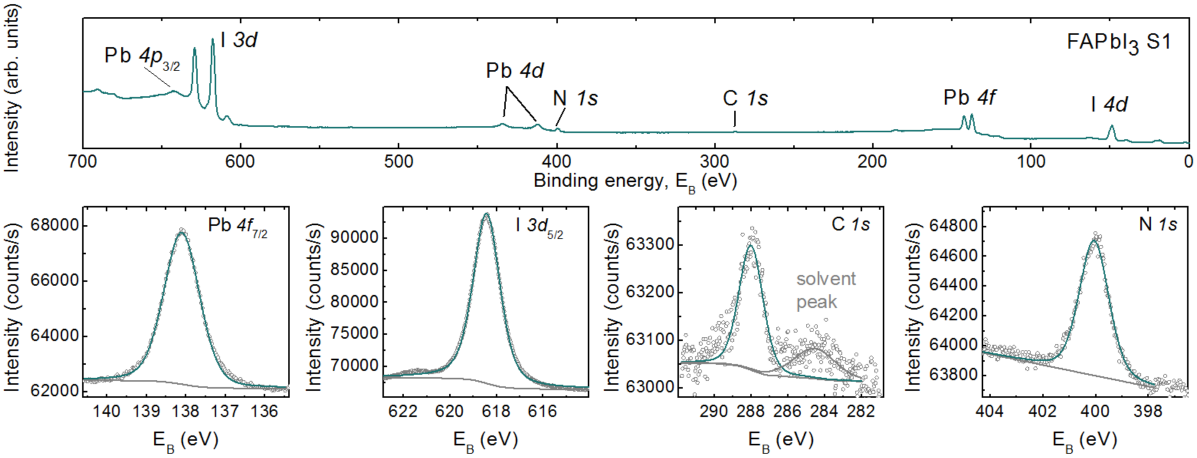

XPS measurement of $FAPbI_3$, sample 1, showing a survey spectrum on top as well as detailed scans of perovskite specific core level signals at the bottom; the solid lines are fits to the measurement (open circles).

Short discussion: XRD shows the well-known tetragonal crystal structure of this black $FAPbI_3$ phase. SEM measurements show close packed crystallites similar in morphology to $MAPbI_3$. XPS shows all expected oxidation states for the elements with some additional more neutral carbon species (likely solvent remaining in the film). UV-vis yields an optical gap of 1.51 eV.

Supplementary Notes 13: $CsPbBr_3$

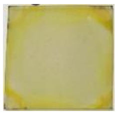

XRD measurement:

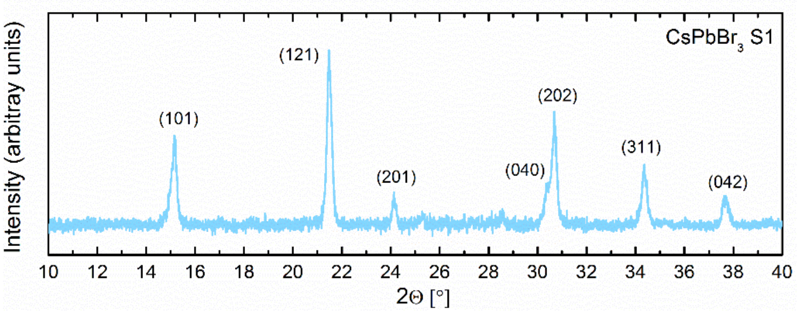

Orthorhombic crystal structure

a = 8.31 Å
b = 11.79 Å
c = 8.21 Å

XRD measurement of $CsPbBr_3$, sample 1; reflexes of the orthorhombic structure are marked and extracted lattice constants are displayed on the right.

SEM:

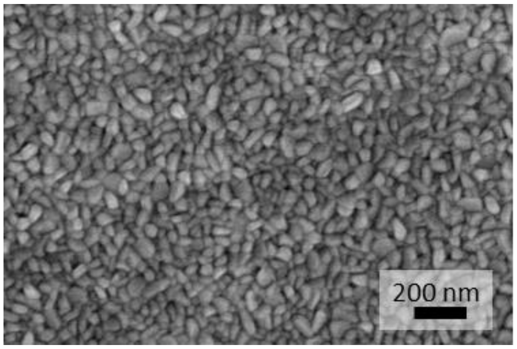

SEM image of $CsPbBr_3$, sample 1.

Absorption:

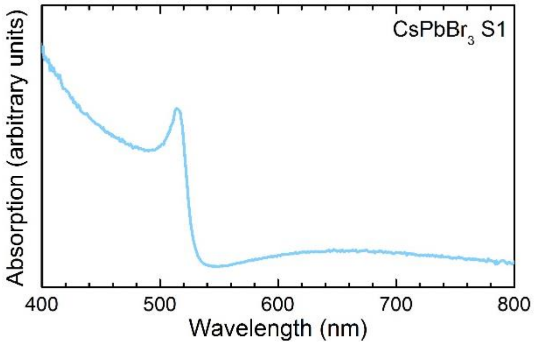

UV-vis measurement of $CsPbBr_3$, sample 1.

XPS analysis:

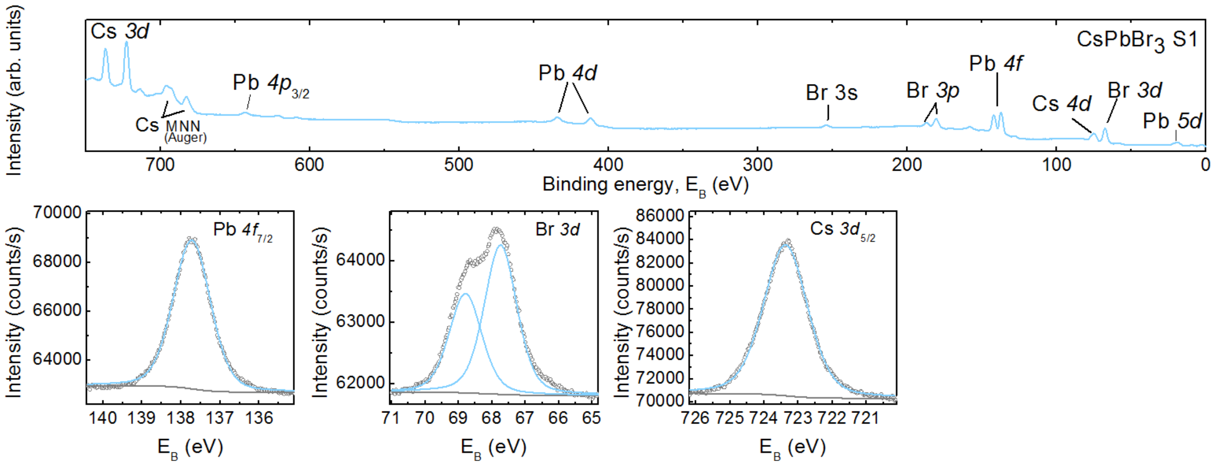

XPS measurement of $CsPbBr_3$, sample 1, showing a survey spectrum on top as well as detailed scans of perovskite specific core level signals at the bottom; the solid lines are fits to the measurement (open circles).

Short discussion: In XRD we find that solution processed films have a preferred orientation ((h00) reflexes missing), while evaporated films are more randomly orientated (not shown). Overall, an orthorhombic crystal structure is found. SEM measurements show close packed crystallites. XPS peak analysis shows the expected oxidations states while UV-vis yields an optical gap of 2.34 eV.

Supplementary Notes 14: $MAPbBr_3$

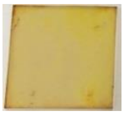

XRD measurements:

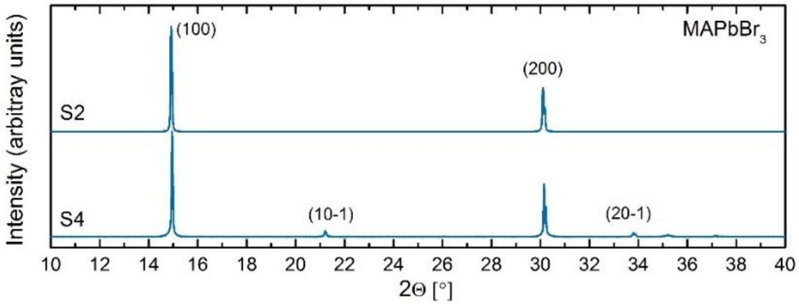

Cubic crystal structure

a = 5.92 Å

XRD measurements of $CsPbBr_3$, samples 2 and 4; reflexes of the cubic structure are marked and the extracted lattice constant is displayed on the right.

SEM:

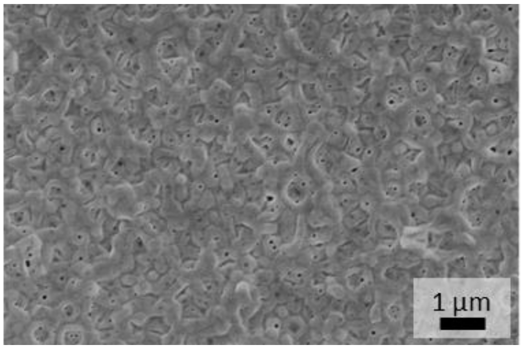

SEM image of $MAPbBr_3$, sample 1.

Absorption:

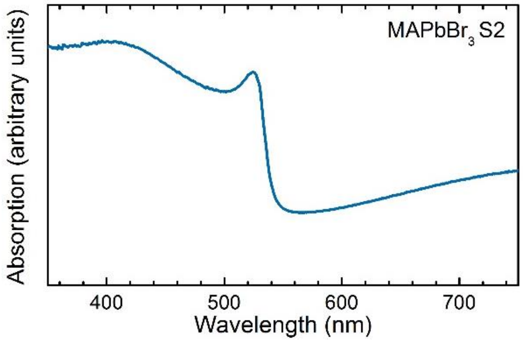

UV-vis measurement of $MAPbBr_3$, sample 2.

XPS analysis:

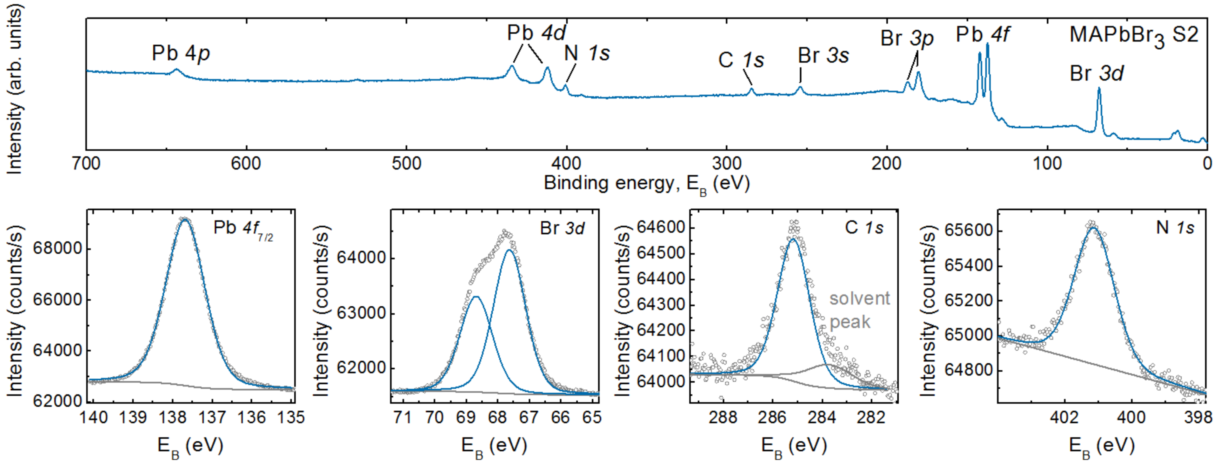

XPS measurement of $MAPbBr_3$, sample 2, showing a survey spectrum on top as well as detailed scans of perovskite specific core level signals at the bottom; the solid lines are fits to the measurement (open circles).

Short discussion: XRD shows the well-known cubic crystal structure, usually with a high degree of order so that only the (100) and (200) reflexes show. A second sample, shown in the image above, also has some weak features from additional reflexes. SEM measurements reveal densely packed films even though the crystallites are not as pronounces as in the case of the *A*PbI_3 compounds. XPS only shows the expected oxidation states with a small additional more neutral carbon species (likely solvent remaining in the film). UV-vis yields an optical gap of 2.28 eV.

Supplementary Notes 15: $FAPbBr_3$

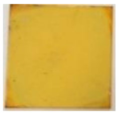

XRD measurement:

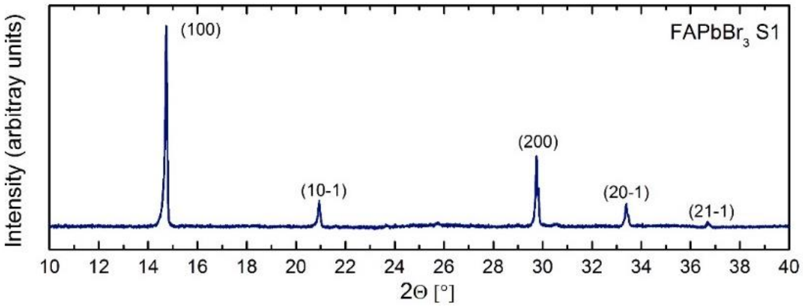

Cubic crystal structure

a = 6.00 Å

XRD measurement of $FAPbBr_3$, sample 1; reflexes of the cubic structure are marked and the extracted lattice constant is displayed on the right.

SEM:

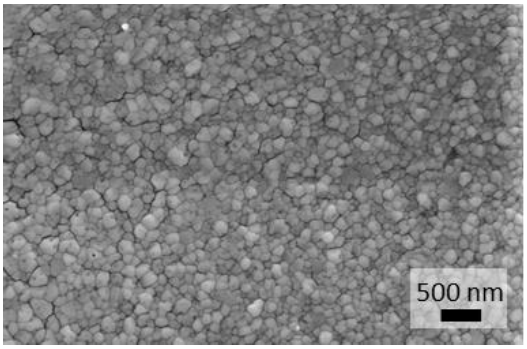

SEM image of $FAPbBr_3$, sample 2.

Absorption:

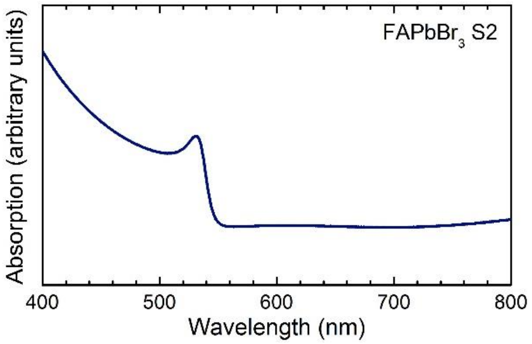

UV-vis measurement of $FAPbBr_3$, sample 2.

XPS analysis:

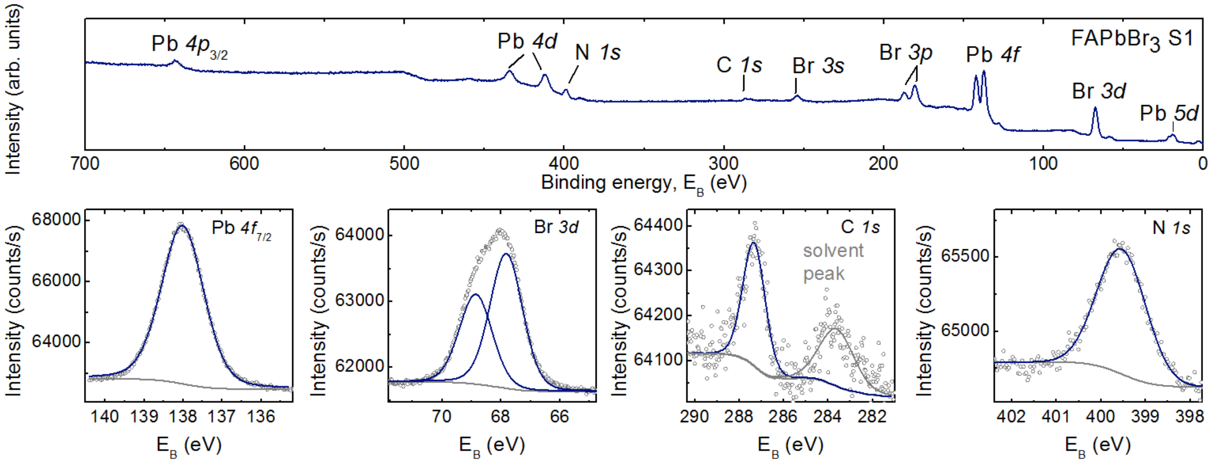

XPS measurement of $FAPbBr_3$, sample 1, showing a survey spectrum on top as well as detailed scans of perovskite specific core level signals at the bottom; the solid lines are fits to the measurement (open circles).

Short discussion: XRD measurements indicate a cubic crystal structure, which has also been observed in powder diffraction measurements in literature [19]. SEM measurements show densely packed films with small crystallites. XPS shows all expected oxidation states for the elements with some additional more neutral carbon species (likely solvent remaining in the film). UV-vis yields an optical gap of 2.26 eV.

Supplementary Notes 16: $CsPbCl_3$

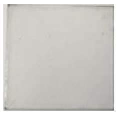

XRD measurement:

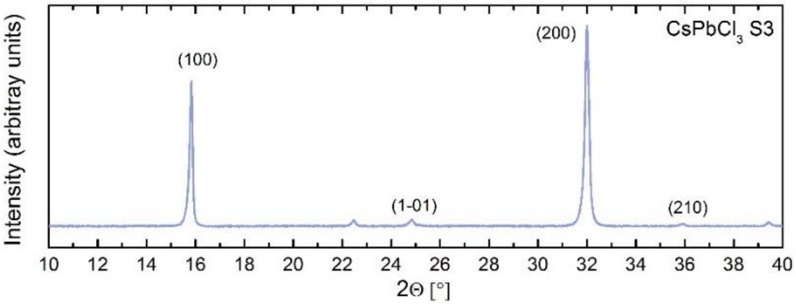

Cubic crystal structure

a = 5.60 Å

XRD measurement of $CsPbCl_3$, sample 3; reflexes of the cubic structure are marked and the extracted lattice constant is displayed on the right.

SEM:

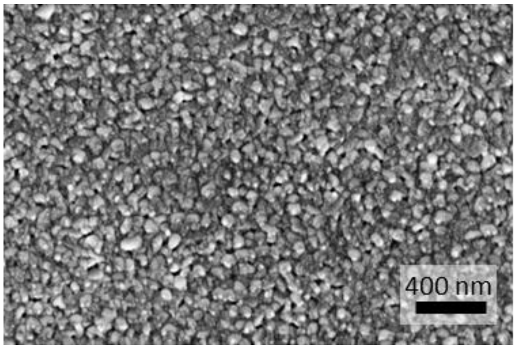

SEM image of $CsPbCl_3$, sample 1.

Absorption:

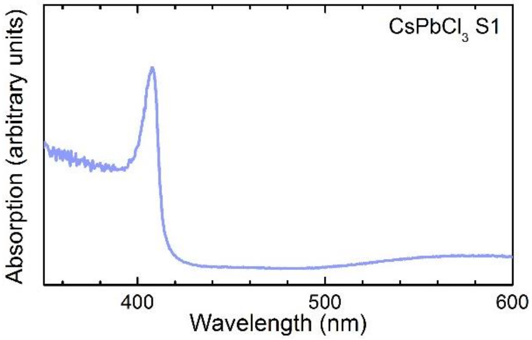

UV-vis measurement of $CsPbCl_3$, sample 1.

XPS analysis:

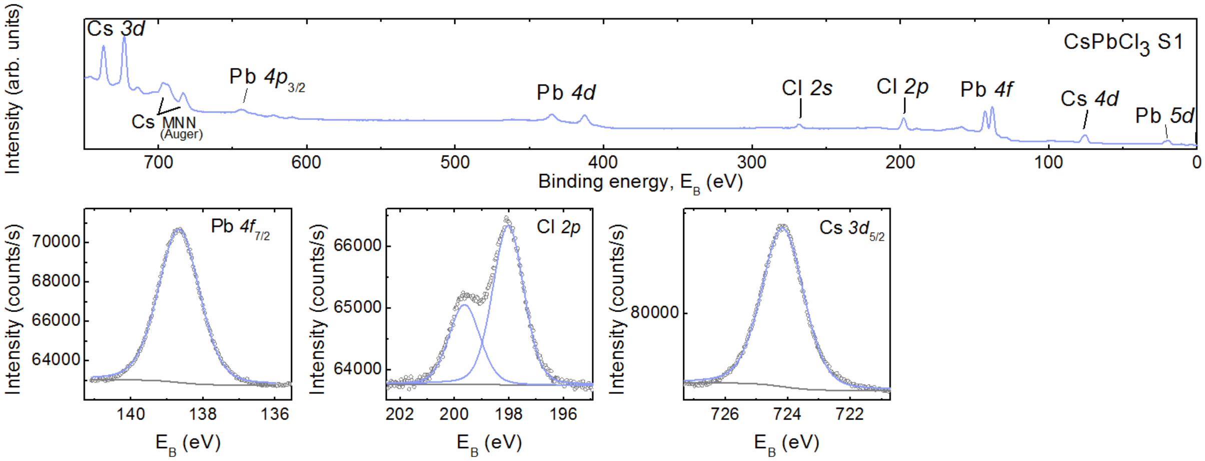

XPS measurement of $CsPbCl_3$, sample 1, showing a survey spectrum on top as well as detailed scans of perovskite specific core level signals at the bottom; the solid lines are fits to the measurement (open circles).

Short discussion: All samples had to be prepared by vacuum evaporation, since CsCl is not soluble enough in commonly used solvents. XRD measurements indicate a cubic structure, which is in agreement with published powder measurements [20]. SEM measurements show close packed films, with relatively small crystallites, as often observed for vapor deposition. XPS shows the expected oxidation states for all elements. UV-vis yields an optical gap of 2.98 eV.

Supplementary Notes 17: $MAPbCl_3$

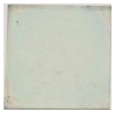

XRD measurements:

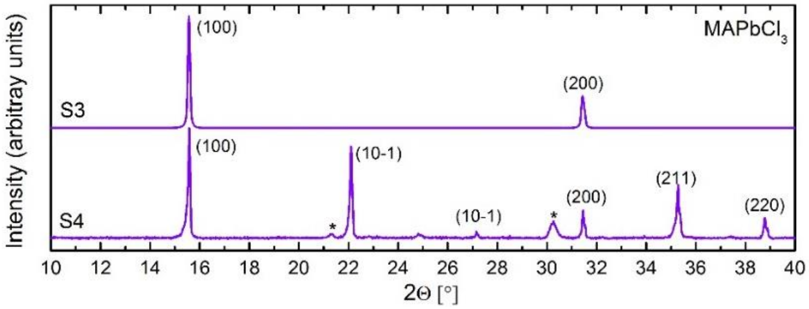

Cubic crystal structure

a = 5.69 Å

*XRD measurements of $MAPbCl_3$, samples 3 and 4; reflexes of the cubic structure are marked and the extracted lattice constant is displayed on the right. Peaks marked by * in sample 4 originate from the ITO substrate.*

SEM:

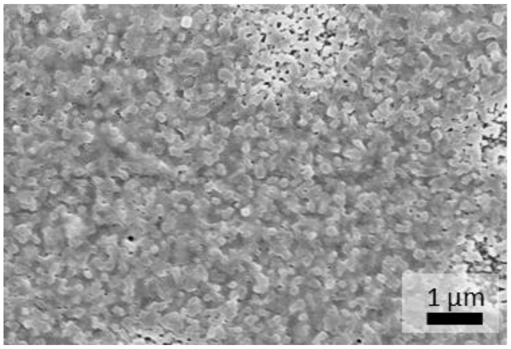

SEM image of $MAPbCl_3$, sample 1.

Absorption:

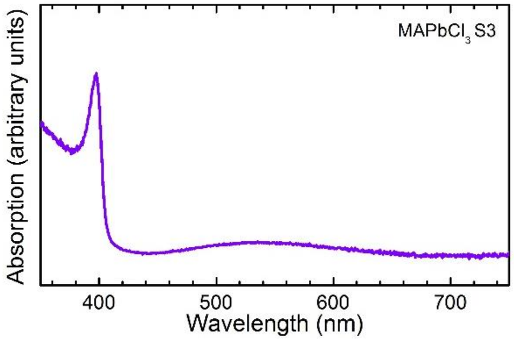

UV-vis measurement of $MAPbCl_3$, sample 3.

XPS analysis:

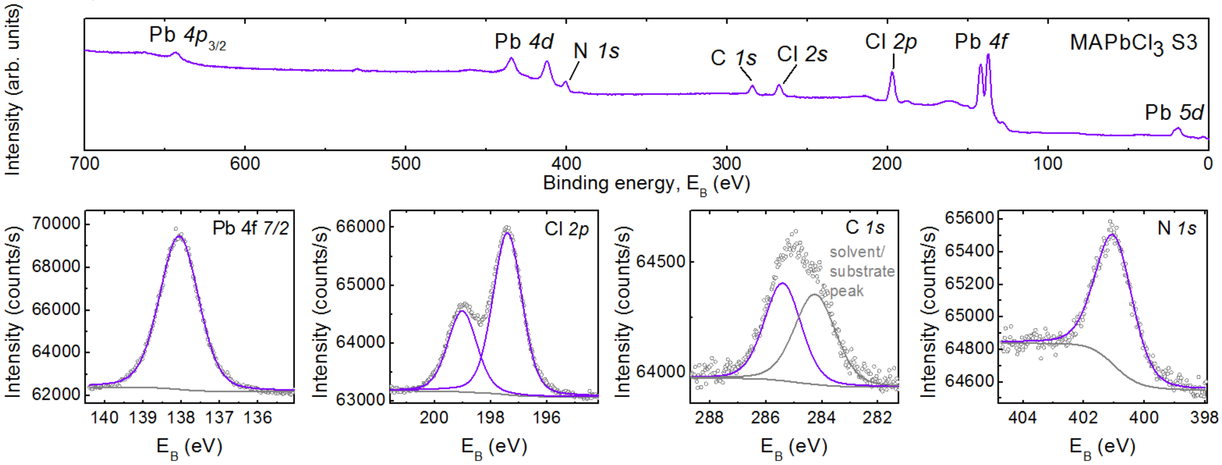

XPS measurement of $MAPbCl_3$, sample 3, showing a survey spectrum on top as well as detailed scans of perovskite specific core level signals at the bottom; here the solid lines are fits to the measurement (open circles).

Short discussion: XRD measurements indicate a cubic crystal structure, which is in agreement to published powder data [21]. SEM measurements show a densely packed film with a few small pinholes and irregularly shaped crystallites, quite similar to $MAPbBr_3$. XPS shows the expected oxidation states, however with a significant additional contribution for C1s. Here, it is likely not only solvent remaining in the film, but in addition the underlying PEDOT:PSS layer is showing through the pinholes. UV-vis yields an optical gap of 3.05 eV.

Supplementary Notes 18: $FAPbCl_3$

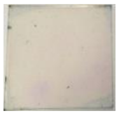

XRD measurement:

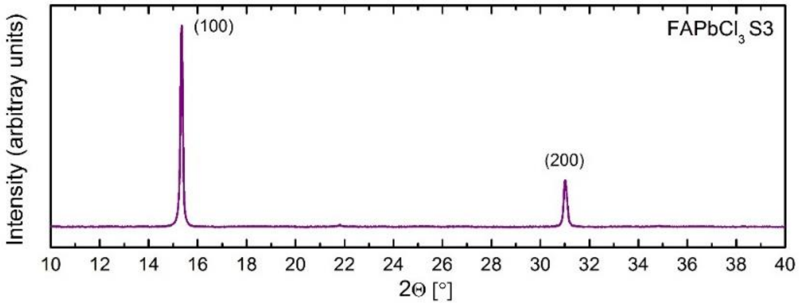

Cubic crystal structure

a = 5.77 Å

XRD measurement of $FAPbCl_3$, sample 3; reflexes of the cubic structure are marked and the extracted lattice constant is displayed on the right.

SEM:

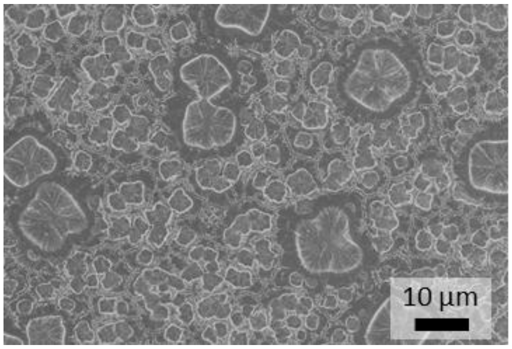

SEM image of $FAPbCl_3$, sample 1.

Absorption:

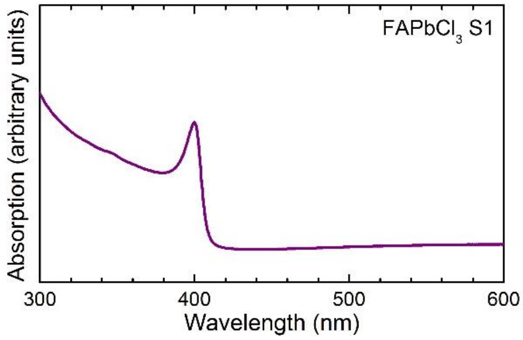

Fig b UV-vis $FAPbCl_3$ S1.

XPS analysis:

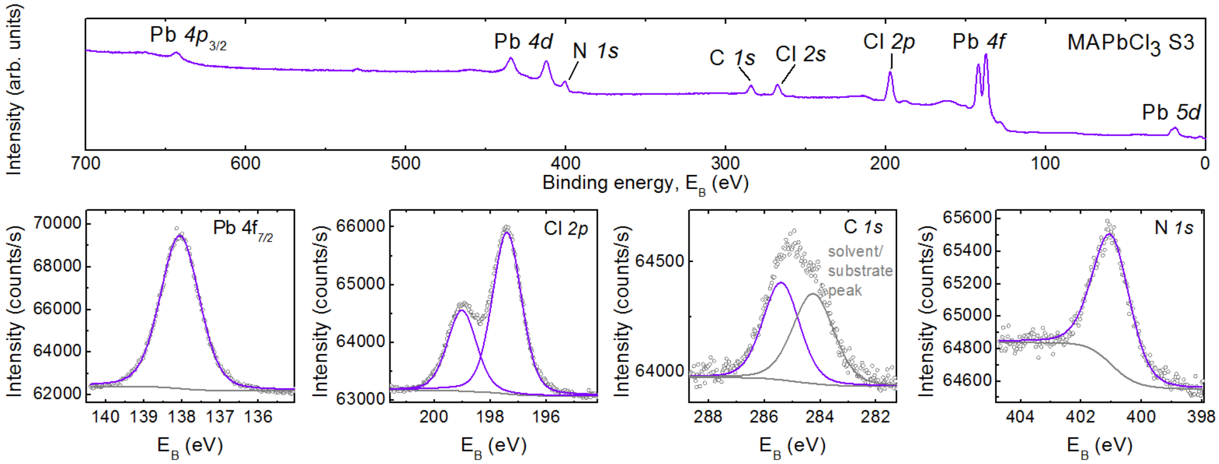

XPS measurement of $FAPbCl_3$, sample 2, showing a survey spectrum on top as well as detailed scans of perovskite specific core level signals at the bottom; here the solid lines are fits to the measurement (open circles).

Short discussion: XRD indicates a cubic crystal structure, which has been reported before for single crystals at RT [22]. SEM images reveal that film formation is not as good as for other lead based systems. Rather large "flower like" features show on the surface, leading to significant parts of the surface not being covered. This is obvious as well in XPS, where a strong contribution of C 1s at lower binding energy is observed, which originates from the underlying PEDOT:PSS layer. The perovskite related peaks show the expected oxidation states. UV-vis yields an optical gap of 3.02 eV.

4. Supplementary Discussion

Experimental sample-to-sample variation

The samples for each material shown above in Supplementary Figure 6 have been selected based on various quality considerations, e.g. XPS stoichiometry, XRD signal, and the correct position of Cs/MA/FA related features (as discussed for Figure 1 in the main article); these "good" films, therefore, show only little sample-to-sample variation. However, there can be significant variations in the shape of the DOS if the stoichiometry is not met. This will lead to deviations from the calculated DOS due to (i) signals present from additional material phases, or (ii) presence of interstitial or defect states; (iii) in addition, measurements by PES can contain artifacts due to the fact that here only the surface is measured, which will electronically differ to some degree from the bulk and can be determined by the specific surface termination. While the individual contributions of (i) to (iii) are difficult to quantify here, we would like to show some examples where the stoichiometry (as determined by XPS) deviates from the expected one either due to overall off-stoichiometry or the accumulation of certain phases at the surface.
Three examples are presented, with different degrees of sample-to-sample variation; these "bad" samples are of course not included in the data evaluation throughout this work but help here to identify good film composition.
How much the features at the VBM and CBM were found to be affected by composition strongly depended on the material. For example, $CsSnI_3$, $MASnI_3$, $MASnCl_3$, $CsPbBr_3$, and $MASnBr_3$ were quite sensitive, while $MAPbI_3$, $FAPbI_3$, $CsPbI_3$, $FASnI_3$, $CsSnCl_3$, or $FASnBr_3$ were much less affected in the band onset region by preparation conditions.

A) Example of $CsSnI_3$
In order to compare the different perovskite spectra here and in the following two examples, the measurements have been shifted such that the VB features overlap; this way variations in Wf are eliminated and the position of E_F is arbitrary. The first example, presented in Supplementary Figure 7, is $CsSnI_3$ which shows one of the most extreme variations in both VB onset region and unoccupied DOS. Samples with additional CsI (as observed by XPS) show additional DOS in the region between 4 and 8 eV. Comparing this with pure CsI spectrum (red curve) it is clear that this is simply the DOS of CsI appearing. At the same time the high binding energy cutoff shifts to the left, meaning that the IE decreases (since spectra are aligned at VB edge).

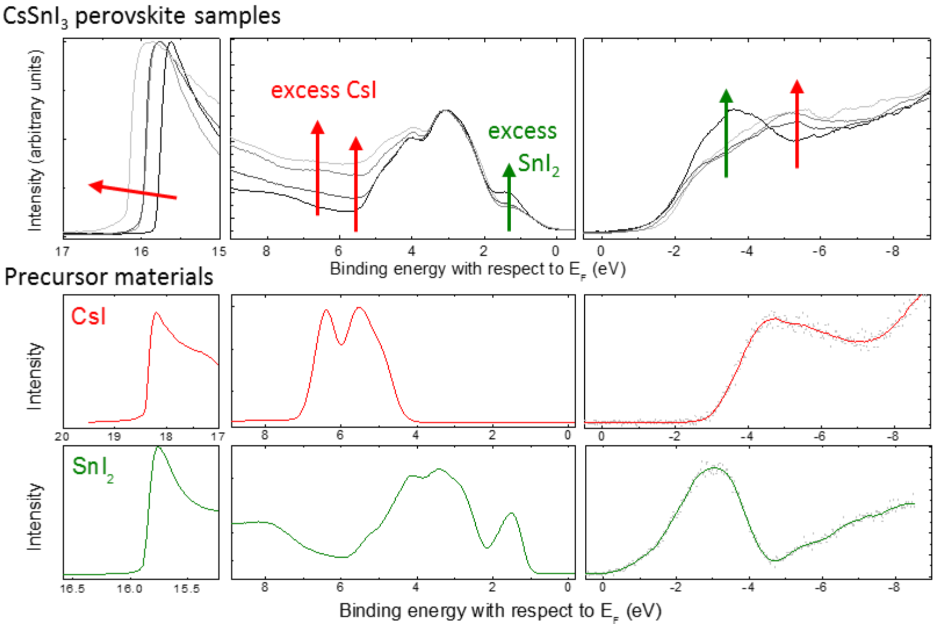

Supplementary Figure 7 | Examples of sample-to-sample variation in UPS/IPES measured DOSs for $CsSnI_3$, where also samples with "bad" stoichiometry are included (upper panel). The two bottom graphs show measurements of the pure precursor materials (CsI and SnI_2) used for this perovskite preparation.

With excess SnI_2 on the other hand, a more pronounced DOS at the VB onset is observed. This most likely originates from the strong VB feature between 1 and 2eV of an additional SnI_2 phase, as seen in the bottom spectrum (green). The surface of $CsSnI_3$ seems to be extremely sensitive, switching easily from CsI rich to SnI_2 rich due to minor changes in composition. For the unoccupied DOS, measured by IPES, we find a strong influence as well. Probably the feature between -2 and -4 eV originates from the SnI_2 and vanishes for the CsI rich films.

B) Example of $MAPbI_3$

Sample-to-sample variations for $MAPbI_3$ are shown in Supplementary Figure 8. Here, changes close to the band onsets are not very pronounced even though again the Wf changes, meaning that the IE changes as well. The overall similarity of the "good" and "bad" stoichiometry films could be due to the fact that $MAPbI_3$, MAI, and PbI_2 all have somewhat similar shapes of the VB region and also in the CB region $MAPbI_3$ and PbI_2 are difficult to distinguish. More pronounced changes are found deeper in the band, around 4 to 7 eV in the VB region and -4 to -7 in the CB region. Here, with excess MAI the DOS increases which can be correlated to features in the pure MAI spectrum; these indicate therefore that excess MAI is present.

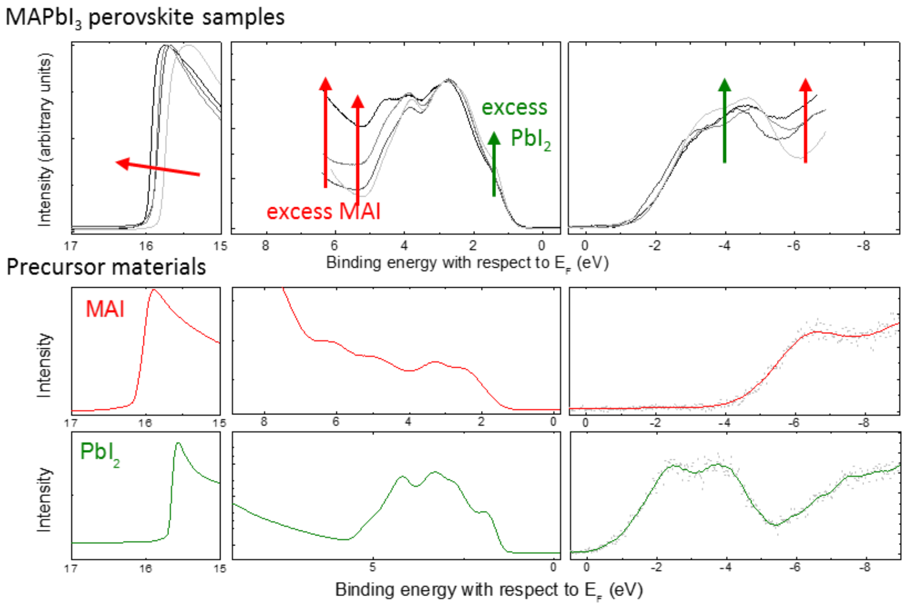

Supplementary Figure 8 | Examples of sample-to-sample variation in UPS/IPES measured DOSs for $MAPbI_3$, where also samples with "bad" stoichiometry are included (upper panel). The two bottom graphs show measurements of the pure precursor materials (MAI and PbI_2) used for this perovskite preparation.

C) Example of $MAPbCl_3$

Finally, in Supplementary Figure 9 $MAPbCl_3$ is shown. Since the solubility of $PbCl_2$ is limited, no sample ever showed an excess in $PbCl_2$ content. However, we had a large number of samples with different degrees of MACl excess which are shown here. Strong variations around 4eV are observed in the occupied DOS, which must originate from excess MACl material that has a pronounced feature here. The DOS of $PbCl_2$ shows characteristic features deeper in the bands, around 5 to 6 eV and -6 to -8 eV, where variations can also be found. Similar to the previous two examples, with increasing amount of MACl the high binding energy cutoff shifts, resulting in a decrease in IE.

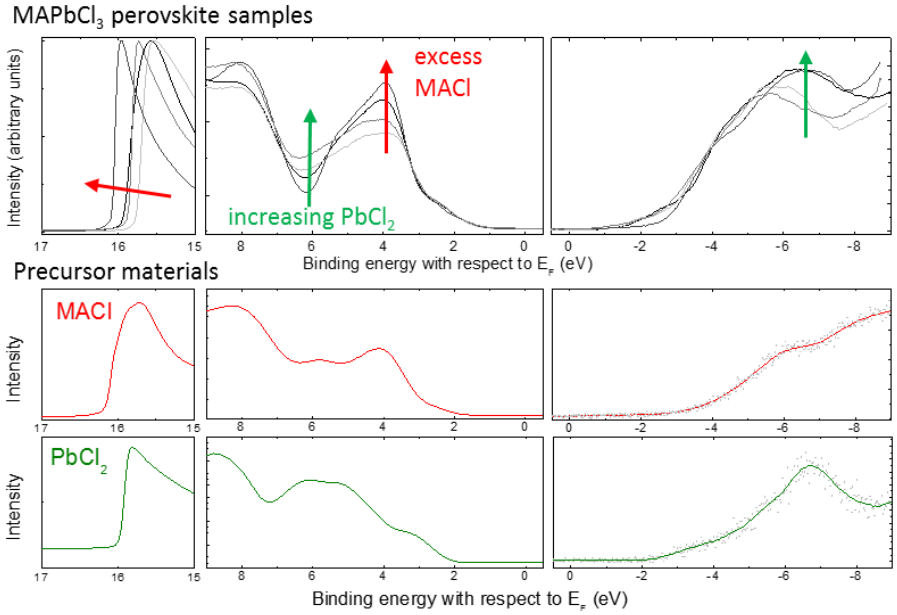

Supplementary Figure 9 | Examples of sample-to-sample variation in UPS/IPES measured DOSs for $MAPbCl_3$, where also samples with "bad" stoichiometry are included (upper panel). The two bottom graphs show measurements of the pure precursor materials (MACl and $PbCl_2$) used for this perovskite preparation.

Changes in ionization energy with different compositions

As indicated for the three examples above, the high energy cutoff changes with respect to the VB onset when the composition is varied, indicating a change in ionization energy, as we have previously reported in a detailed study for $MAPbI_3$ [12]. Throughout this study we noticed that some perovskite compounds show stronger variations that others probably indicating that their surface composition is more easily affected. Even though no systematic variation was done, we present the corresponding data in Supplementary Figure 10, in order to show the interested reader the trends for the various compositions. It should be noted that for this plot the ionization energy was read out by a simple linear fit to the slope of the VB DOS, therefore values at the correct stoichiometry of 1 differ somewhat from the ones reported in Table 1 and Figure 5 of the main article, where the more elaborate DFT based fitting procedure was employed.

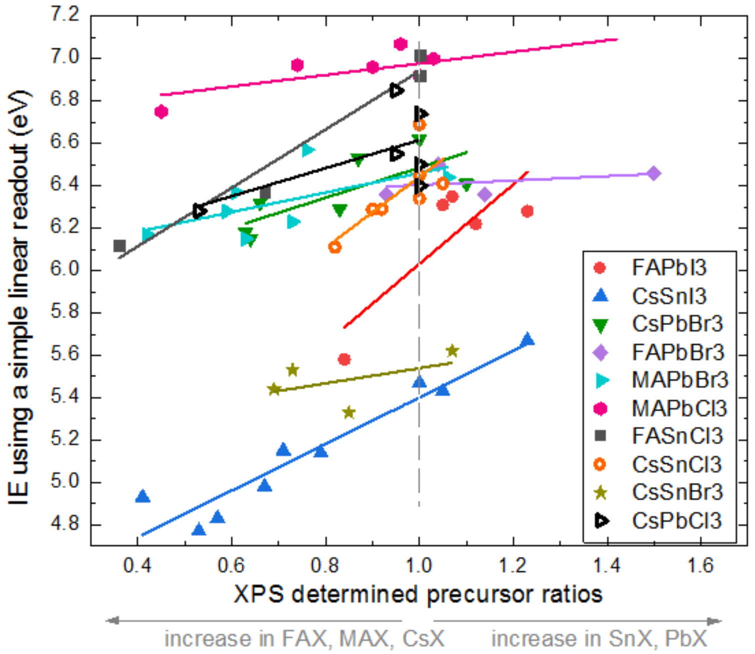

Supplementary Figure 10 | General overview over variations in IE due to unintentional variations in sample stoichiometry. Note that these data points were obtained throughout the optimization process of sample fabrication and samples with off-stoichiometry were not used to determine the IE and EA values presented in the main article.

5. Supplementary References

1. Amat, A. *et al.* Cation-induced band-gap tuning in organohalide perovskites: Interplay of spin-orbit coupling and octahedra tilting. *Nano Lett.* **14,** 3608–3616 (2014).
2. Kang, J. & Wang, L. W. Dynamic Disorder and Potential Fluctuation in Two-Dimensional Perovskite. *J. Phys. Chem. Lett.* **8,** 3875–3880 (2017).
3. Ma, J. & Wang, L. W. Nanoscale charge localization induced by random orientations of organic molecules in hybrid perovskite CH3NH3PbI3. *Nano Lett.* **15,** 248–253 (2015).
4. Zhu, H. *et al.* Organic Cations Might Not Be Essential to the Remarkable Properties of Band Edge Carriers in Lead Halide Perovskites. *Adv. Mater.* **29,** 1–6 (2017).
5. Yaffe, O. *et al.* Local Polar Fluctuations in Lead Halide Perovskite Crystals. *Phys. Rev. Lett.* **118,** 136001 (2017).
6. Tao, S. X., Cao, X. & Bobbert, P. A. Accurate and efficient band gap predictions of metal halide perovskites using the DFT-1/2 method: GW accuracy with DFT expense. *Sci. Rep.* **7,** 14386 (2017).
7. Davies, C. L. *et al.* Bimolecular recombination in methylammonium lead triiodide perovskite is an inverse absorption process. *Nat. Commun.* **9,** 293 (2018).
8. Endres, J. *et al.* Valence and Conduction Band Densities of States of Metal Halide Perovskites: A Combined Experimental–Theoretical Study. *J. Phys. Chem. Lett.* **7,** 2722–2729 (2016).
9. Bao, J. L., Gagliardi, L. & Truhlar, D. G. Self-Interaction Error in Density Functional Theory: An Appraisal. *J. Phys. Chem. Lett.* **9,** 2353–2358 (2018).
10. Komesu, T. *et al.* Surface Electronic Structure of Hybrid Organo Lead Bromide Perovskite Single Crystals. *J. Phys. Chem. C* **120,** 21710–21715 (2016).
11. Philippe, B. *et al.* Valence Level Character in a Mixed Perovskite Material and Determination of the Valence Band Maximum from Photoelectron Spectroscopy: Variation with Photon Energy. *J. Phys. Chem. C* **121,** 26655–26666 (2017).
12. Emara, J. *et al.* Impact of Film Stoichiometry on the Ionization Energy and Electronic Structure of CH 3 NH 3 PbI 3 Perovskites. *Adv. Mater.* **28,** 553–559 (2016).

13. Dang, Y. *et al.* Formation of Hybrid Perovskite Tin Iodide Single Crystals by Top-Seeded Solution Growth. *Angew. Chemie - Int. Ed.* **55,** 3447–3450 (2016).
14. Chiarella, F. *et al.* Preparation and transport properties of hybrid organic–inorganic CH3NH3SnBr3 films. *Appl. Phys. A* **86,** 89–93 (2006).
15. Ferrara, C. *et al.* Wide band-gap tuning in Sn-based hybrid perovskites through cation replacement: The FA1-:XMAxSnBr3mixed system. *J. Mater. Chem. A* **5,** 9391–9395 (2017).
16. Scaife, D. E., Weller, P. F. & Fisher, W. G. Crystal preparation and properties of cesium tin(II) trihalides. *J. Solid State Chem.* **9,** 308–314 (1974).
17. Chiarella, F. *et al.* Combined experimental and theoretical investigation of optical, structural, and electronic properties of CH3NH3SnX3 thin films (X=Cl,Br). *Phys. Rev. B - Condens. Matter Mater. Phys.* **77,** 045129 (2008).
18. Sutton, R. J. *et al.* Cubic or Orthorhombic? Revealing the Crystal Structure of Metastable Black-Phase CsPbI3by Theory and Experiment. *ACS Energy Lett.* **3,** 1787–1794 (2018).
19. Zhumekenov, A. A. *et al.* Formamidinium Lead Halide Perovskite Crystals with Unprecedented Long Carrier Dynamics and Diffusion Length. *ACS Energy Lett.* **1,** 32–37 (2016).
20. Sebastian, M. *et al.* Excitonic emissions and above-band-gap luminescence in the single-crystal perovskite semiconductors CsPbB r3 and CsPbC l3. *Phys. Rev. B - Condens. Matter Mater. Phys.* **92,** 1–9 (2015).
21. Baikie, T. *et al.* A combined single crystal neutron/X-ray diffraction and solid-state nuclear magnetic resonance study of the hybrid perovskites CH3NH3PbX3 (X = I, Br and Cl). *J. Mater. Chem. A* **3,** 9298–9307 (2015).
22. Govinda, S. *et al.* Critical Comparison of FAPbX3and MAPbX3(X = Br and Cl): How Do They Differ? *J. Phys. Chem. C* **122,** 13758–13766 (2018).
